# Supplementary material for: Community health volunteers’ experiences during the COVID-19 pandemic in Kiambu county, Kenya: A qualitative study
Source: PLoS One. 2025 May 7;20(5):e0322642. doi: 10.1371/journal.pone.0322642 (PMC12057936; doi:10.1371/journal.pone.0322642)
Supplement: S1 File — This document contains both the audio transcriptions of the focus group discussions translated into English. (PDF) [file pone.0322642.s001.pdf]

Interviewer: And our first question is: kindly share your experiences during the period of the COVID-19 lockdown.

Respondent 1: the experience I had was that, first of all, I am from Limuru sub-county and that is where the COVID centre is located. We closed our CCC, our triage and our clinics. People stopped coming to the clinics, and it affected us who worked at these clinics such as the CCC, diabetic clinics, PNC, all were closed down. People struggled a lot, if they came to Limuru, there was a roadblock in [unintelligible], another one was in Siada, another one was in Lari, another one was in Mtarakwa. You wouldn't be able to reach the hospital. It forced people into not receiving services from the hospital because even if you went to the DO there at the Health Centre there was no clinics or doctors at those clinics. There were no facilities either, not many. Therefore, people struggled a lot that is my experience during COVID-19 at the community level. we struggled so much. even right now when they come to the clinic like CCC because we have opened, we keep calling them but they have left. The say they have gone to Nairobi and they can't come because of the roadblocks. Many, many people were lost to follow up at the clinics, even the TB clinics. Therefore, we have already gotten many problems due to lockdowns and COVID-19. Yeah.

Interviewer: anybody else who would want to share their experiences with COVID-19? I believe we had many experiences so please share.

Respondent 2: even here at Kiambu there are not many people who come. Even when they come there is not anyone who is getting treated. They are scared of COVID, therefore even all the facilities don't have any people. I don't know where they're going, they're just staying with their illnesses. Even the people with TB are now bed ridden. So, we had many problems with COVID.

Interviewer: so, was it that time or even now?

Respondent 2: Even now they're scared of coming to the facility. Even that time they were afraid even those ones who are very sick with other illnesses are scared of coming to the No no

Respondent 3: and right now, it's not just that they are scared of coming to the facilities it is also because they don't have money. Some of them don't even have fare. Some of them should be getting their blood pressures and their sugar levels checked but they say they don't even have fare to reach the hospitals. There is no money. Yeah.

Respondent 4: most of the people who had been employed were got fired after COVID hit. So, most of them went home and then there were many problems when COVID came because most of the people didn't have income, they

did not have money. People we had been following for TB treatment stopped coming for the treatment and some of the defaulters just went home. Even right now they have not resumed treatment because they just feel that COVID is there. So right now, we are trying to get them back the ones that we can bring back to the hospital. Thank you.

Interviewer: **were you able to carry out your normal activities as the community health volunteers during the COVID-19 lockdown?**

Respondent 1: no, we were not able to carry out because first of all we were not more creative with COVID. We were not aware we were just told things about COVID. We didn't have that experience so even the data have we didn't have people going to collect the data because we were scared. We were told to stay at home. So, you couldn't go anywhere, you were just sat at home. And the important thing is that our prayers were answered as we were called once in a while to be taught about COVID. And then we could talk to people about COVID as long as they were socially distanced. You could write down what you wanted or you could refer them to someone else. The patient could tell you that they were feeling like this or like that and you could refer them to someone else but before this we did not have that experience. Right now, we all have that experience but before we got the experience many of us were very scared. But before we had problems because the patient would say that they have an illness and they need to go to hospital but we didn't know which hospital to send them to, and at the same time they were also scared. The mothers were scared to attend ANC clinic and at the same time they were also scared of getting their children immunized, even diabetics were scared to go and get their medicine because they could also get COVID. so, we were very scared. We had many problems at that point.

Respondent 2: we would train for COVID for two weeks… so we would talk about COVID and other illnesses. we were trained for COVID and home-based training a bit a bit here and there. But the problem we started to experience was that no one wanted to open up and when you told them you could go to the clinic they did not want to go. They would just refuse to go to the clinic even if you offered them help to go to the clinic. a patient of mine even had two of her children die, some of them are diabetics and some of them have high blood pressures and they would completely refuse they would say 'we will go there to die'. Even another one died as she was giving birth they were scared and completely refused. In fact, another time I went to a house and they asked me what have I brought for them, have I brought for them COVID? I felt very bad but I just went on. This disease has destroyed everything even the economy. No one would open up, and we didn't get any gloves we didn't get any masks we didn't get any anything. You would really apply yourself to your job

and there was a time I even went to donate food because there was a family that said corona has really messed up their income. God has helped but there were very many challenges very very very many challenges. We just used to apply ourselves completely to a job and regardless God has helped. It is a problem but it will come to an end. Yes.

Interviewer: **so how did you overcome the challenges… the challenges that you faced during lockdown regarding your duties?**

Respondent 1: in the beginning it was all happening all of a sudden, we didn't know what our lockdown was we didn't know what COVID was. Therefore, we attended two or three seminars to get educated on the matter and then when we returned to our hospitals, we were given PPEs such as masks and etc. And then when we went back to the community, we had to communicate with them at the door or a bit further away from their homestead, we could not enter their rooms. During the seminars we learned how to talk and to the mothers for ANC clinics, we also learned how to deal with the diabetics. We did have some challenges such as the diabetics not having any medication but since they knew which medications, they were taking we took the responsibility to go to the hospitals and get a two week or a one-month supply of their medication and give it to them at their house because they were not going to the clinics/hospitals. So, we were working very closely with the facilities and they helped us out a lot. And at least we got some knowledge about COVID and how we would tackle the arising issues. Yes. Thank you.

Interviewer: **Thank you.**

Respondent 2: what I can add is that the challenges we overcame. Us we do report to Wanginge Medical Centre and we had a lot of trainings. And these trainings helped us out a lot and everywhere the message was sent it was like a song it was even in the radio even children school going children would understand the message distance wash your hands sanitize everything so that more people the more people learn about social distancing, washing hands, everywhere there was water. So, if you enter a house, you were told first to wash your hands before you enter the house. and before you enter the house, you're also told let's keep social distance. and now that is what helped us to overcome such challenges. Yes.

Respondent 3: yeah, we had one child that fell sick and due to the stigma that child could not even leave their home. We used to have seminars and since the community didn't even want to be associated with them during the seminars, we had to visit that child ourselves and since the community saw that we were going to their house since they had gotten well is

when they realized that one could recover from COVID and that you could visit someone who had the disease and not get it yourself. We even went to visit the school and said COVID is there but let this child attend school with the other children. then some stigma was lifted and people began to associate with those who had the disease but still some people kept off. So, I think that one – even us our work started to go back to normal. We could enter people's houses and say that COVID is there, wash your hands, sanitize, children wear masks, like that.

Interviewer: **Thank you. In your opinion, how did the community respond to their COVID-19 measures?**

Respondent 1: initially people were very apparent to the COVID-19 guidelines such as washing their hands regularly wearing masks keeping social distance and staying at home. But eventually they stopped listening to the stay-at-home orders because there was no job there was no food and there was no nothing at home. Also, with the children at home some of them would play with the mask and others would not so if there were ten children you would find five of them or playing without a mask on. But eventually the notion was that this is a disease that is here to stay and it is a disease we have to live with. And if such is the case, why should they stay at home? why should they wear a mask? why should they lock themselves in their home? why shouldn't they go to work? why shouldn't the children go… you see? And it was expected that the government would provide their basic needs but then you aren't at work the kiosk isn't open and, in the evening, you have to eat something. So quite a number of them began to stop adhering to the stay-at-home orders. And then we had this issue of roadblocks. people would travel from out of the county, from let's say Kisumu, and they would have met the roadblocks somewhere in Lari and then they would go using a motorbike. So, if such people were allowed to come in the county and yet we were told that there is COVID but yet they have been allowed by to go to Kisumu or Migori and they have come till here using a motorbike until the roadblock. The question then became is the government serious about these containment measures? You yourself you were disturbed and bothered enough to follow the rules and stay at home but other people are traveling for calls and businesses. Yes.

Interviewer: **thank you. Anyone else who would want to respond to that? How did your community respond to COVID-19?**

Respondent 2: there, the community initially responded, but after a while they began to forget. Because I remember when you would work in the COVID centres you would come out to get a bus but the way people would look at you they would look at you like you are coming from sick people. But eventually they understood how to prevent infection by washing hands,

keeping distance and wearing masks. But even if they did all that there was no money for you to buy food, there were no jobs, GBV increased again, gender-based violence. Because the husband didn't have money and the wife did not have money, so you would just hear people were kicked out of their homes, people were fighting each other because of gender-based violence… there was nothing to eat. But at the same time the community understood things about COVID. However, the only thing is is that people don't wear masks. Like right now we are wearing masks but if you pass by other areas, you will not find a single person wearing a mask. They threw them away. So even right now the community needs sensitization that COVID is still here and we must continue the preventive measures against it. That's it. So, the community responded well, but there are many challenges ahead of us if we don't sensitize properly. Because you see they'll just ignore, they'll just ignore it completely. Yeah. When they enter clubs, they will not social distance, even in bars. See the bars open at 7:00 PM or 8:00 PM they do not social distance. They don't wear masks. It's really hard in some areas, you can enter a place and you won't even have to sanitize, you just enter. There is no machine for temperature, Thermogun, you just enter. So even still we have a challenge, we have a challenge even right now.

Interviewer: **thank you for your inputs.**

Respondent 3: another thing is, for example like in church when you go back to church people have forgotten from time-to-time for example when you enter church it can just be you wearing a mask. People start saying maybe you are sick because you are wearing a mask, everyone else don't think it matters. That thing for measuring [temperature] is not there so you don't know the state of people. When you come out of mass you will find that there's not even any sanitizer, you just enter. It's just you who knows how you will personally enact your preventative measures. If you speak up people will tell you that you have your own issues, some people will understand some people won't understand. So now we say that this is your own responsibility because you know where you have come from and where you are going. Other people say that it won't affect them so they don't have to wear a mask.

Respondent 4: and then for public transport you said that we are not catching many people but then the conductor will ask you 'have you ever heard of her conductor dying of COVID?'. There is also no option to pay through mobile money in a bus and you find that many times that there are more people than is required in the bus. So, in such a circumstance you have to apply your own preventative measures for yourself. Thank you.

Respondent 3: Even right now many people say that there is no such thing as COVID. But in our unit 1 doctor died. So now people wear masks because they think 'that since a doctor has died, what will happen to us? Even us we will die.' Even now when I go to mobilize, people tell me 'We will wear masks now', so my unit is doing better. but not many people wear the masks, only the ones that know COVID exists. thank you.

Respondent 5: what I can say for the community and society at large it is ignorance and corruption. Because like today we left Kabete and we passed five roadblocks with traffic police but our cars were full of people. It all starts with us. Us as CHV community. It's just the way the river flows so if we keep quiet even others will keep quiet and you'll find that the corrupt people are the police number one, the conductors and for instance… can I give an example?

Interviewer: yes, you can.

Respondent 1: it's like facing someone, there are times we are given work at funeral(s) or we cook at funeral(s), you tell someone 'Don't pass here', I also work with the Kenya Red Cross. We are given work. You stand at the gate, there is water and sanitizer. You tell people to wash their hands and sanitize. Others ignore you so then you know everyone goes with their ideas. And then another thing people say there in the community 'the government so they are saying that there is corona so that they get money. Even those cases they'll say 800 have been infected, people have died, so tomorrow they'll say it's at 2000 cases so that they get money. And also, so that they are helped by other countries'… so they say there's nothing. They say it's the government that wants money. Even the other day when Uhuru announced the lockdown, they said 'so, now Kenya doesn't have money so he has locked down, but now the economy has crashed so he'll open things up again.' So that's what people were saying. That's all I have to say.

Interviewer: thank you very much. So, in your opinion, how did the COVID-19 measures affect community access to health facilities? Those measures, those measures that we imposed on us for COVID-19 control. Some of them are like the lockdowns, social distancing, you know, even schools being closed. Hand washing. Sanitizing. How did those measures affect how the community accesses health facilities?

Respondent 1: the problems that have affected us in the community many refused to come to hospital. This is because, this is because, they were afraid. They

were afraid first of all to be assessed. If they see their temperature is getting checked with the thermogun, they get scared, they say 'maybe if it's high I'll be admitted.' OK, others were scared because they were not believing us. First of all, many did not believe that COVID-19 would affect them. They saw it on TV but they didn't know about it well. For them it was political. They saw that it was just political but it really affected the health facilities because people weren't coming to the clinics, they didn't pick up their medicines, they were scared especially if they saw the thermogun, if their temperature was checked they didn't want it to be checked. They said they aren't sick. We used to measure temperature at the facility. If you said…if you said to someone 'come, let me check your temperature' they would tell you 'I'm not sick.' They were really affected. really really really really. Even immunizations, the mothers coming for immunizations… they finished by the way. They would ask 'Are there still injections?' the mothers were asking you. 'Is the hospital still functioning or not?'... They had that stigma. They had it a lot. Especially if they knew you were working in the hospital or that you go to the hospital a lot or that you are based here in the facilities, they stigmatize you a lot. It's not like now, now people are at least. They understand what is COVID. Yes.

Respondent 2: OK the COVID-19 measures, where I'm from, they really affected us a lot. You see like fumigation, that pump for that medicine, everyone would hear it. If they heard it and it was fumigation, even that sound of fumigation, people would not come. No, the facility I'm from, we had cases of doctors getting sick. So, it forced us to close the facility and get fumigation done. Sue the community, when they heard doctors were getting sick and fumigation had been done, they didn't want to come to hospital. Even if you find someone sick at home, they would say 'no, that hospital of yours, I don't want it.' And that person is sick. They are sick. So, people coming to the hospital was a problem because they said that 'I will go with my illness, and I will get Corona from your facilities because even your doctors have been infected with this sickness'. If you tried to refute their claims they would say no, even still you had the pump put the medicine there. And then when they found out the hospital was closed for about 3 days, or let's say one week, they knew that that whole area has corona and so they didn't want to enter. Even if you told to come to the hospital she would say 'no, not at your place. Its better I even go to another place.' Oh, and another thing, this issue of social distancing brought a lot of problems. The place I am from they closed the gate, they brought social distancing outside the gate. So, someone would wait outside the gate for two or three hours and they would get tired. They saw that their turn would never reach. They would get tired. They would either opt to go to a private hospital, or the chemist to buy

over the counter medication and then go back home. So, to enter the hospital was a big problem. So even the watchman at the gate would say only two people would enter at a time, even if there are a hundred people at the gate. So, you would just sit outside the gate you have a child, a sick person, you get angry and you just leave. So, it was a bit challenging. And then again it would create…like if people find in your homestead someone gets infected with COVID, you find that that the person is removed from home to go to hospital, and they know that this person is a person of corona, they would say that this person was brought to your hospital and that's where they got sick. Because they have so-and-so… and maybe they come to hospital and die. Then again, that is a problem. You say to someone let's go to hospital and treat you they would refuse. 'I don't want that hospital. It's better I go to a private hospital or I just stay at home.' Yes.

Interviewer: **thank you. In the… in your opinion, how did the COVID-19 measures affect the community capacity to deal with other priority diseases? How did those COVID-19 control measures affect the community capability to deal with other priority diseases? You know we have many diseases. But we have priority diseases. Right now, COVID-19 is a priority, isn't it? So how did the measures against COVID-19 affect the community capacity to deal with other priority diseases?**

Respondent 1: to answer, like those patients who would come to CCC, many were far away and there were roadblocks. For instance, maybe they're from Lari and they come to the facility in Tigoni, and now there was a road block at Mathore. So now will they really pass through? They don't even have that money. So economically, economically, people were affected a lot. Then again, there at CCC, we reached a point where we didn't even measure pressure because a person would be afraid of placing that machine there – social distance. It's a challenge. Because even if they reach there, you tell them to stay far away. maybe if there are 20 people, they have to sit further away from each other and then again, they are scared of each other. But again, you know, even when COVID was initially talked about they said that those people of HIV, diabetes, pressure – those are the people that are at risk. So, these people were completely lost to follow up. Like someone here said, we were forced to call them personally everyday pleading that they come to the clinic. Some of them were stigmatized, yeah? They don't even want the community members to know that actually they need medications, they don't even want that they themselves have the responsibility, because they have HIV and if they come here, they'll be affected by this virus. 'See I will die? let me just stay.' So, the responsibility fell on us CHVs to remind them that the hospital was still operating. That's among the challenges we had. Fine, again, people with diabetes, you know

diabetics are part of the risk groups of COVID. So that diabetic is scared of coming to the clinic to pick up their medications. Even right now there are some who haven't come back to the facility. They buy over the counter. Even people with blood pressure. They are scared of getting their pressure measured because they think that if they go, they'll get COVID. Yeah, many challenges. People of ANC, the mothers… because I remember there was a place I went for a home visit, the mother told me she hasn't been to the clinic in seven months. Seven months! And she hasn't gone to the clinic. I told her 'Let's go, because these months you don't know the state of your child'. I removed her from her house and we went. I only left her once she was seen at the facility, I told her 'Let's not be afraid, let's go. See even me I will be there, let's go'. Then those women who gave birth, they were maybe in the third month and during the 4th month COVID was announced, so many skipped immunizations. They skipped period because when they were told 'stay-at-home. Lockdown.' They didn't have an otherwise. the hospital they could go to maybe it was a private and they didn't have money. So then there are many challenges faced with COVID. These are just a few, these are just a few. thank you.

Interviewer: **thank you very much.**

Respondent 2: in the villages we CHVs we have a tough job. Because we remove someone from home and refer them to a facility and they are scared of COVID. We had the hard job of convincing them there isn't COVID. If you stay safe during the conditions of COVID you will not get it. So, we had a tough job. Thank you.

Respondent 3: let me answer in reference to your question. I want to answer that, when COVID came, we had a… we were taken for seminars. We studied for eight days about this, about COVID. We were shown how to construct the tippy tap in the community. Something to wash hands. So, we went to homes and taught them how to make it and there was no resistance not even a bit. So, most of the homes - we made it, we showed them, and they made it. Now, since they used these well, we saw that other diseases like diarrhoea went down, these other diseases went down due to hygiene. So, I see it like if they can continue using the way we have showed them in the villages, other diseases will go down like some of them went down completely. They were not even taken to hospital because of hygiene. Thank you.

Interviewer: **thank you. So, what are the community perspectives about COVID, about its origins? Community perspectives of COVID about its origin… this means what did the community think or feel about where COVID came from? Or how did COVID come about. What does the community think about it?**

Respondent 1: the community were very vigilant about this matter. Because remember, in our area there are many Chinese people, the ones who are constructing that road. Now, when community members saw a Chinese person, they would think that that is the person who has brought COVID. Yeah? And then if they got to know that you were abroad, they begin to suspect that you have brought COVID. Remember, when we initially had the lockdown, there were these girls that came from Saudi Arabia, others from India, they came. People called me, 'you, Kariuki, there is this girl who has just come, maybe you should go and check on her so she doesn't come out of her house.' You, see? The community… they were not aware that first of all there isn't anyone who can't get COVID. And it's not a must that if someone comes from abroad that they have COVID. That's what people were doubting, but right now, they say that the people coming with the plane are the ones who have COVID. That's what I used to see, that's what they used to think. Then again if they see a Chinese person, they just assume that they have COVID because they heard it came from, I don't know, Huawei… I don't know, Huawe-…. Now that's what they used to think.

Respondent 2: now before, when COVID just came, they never knew what is COVID. So, many said that it was an illness brought from China he said that it was just for China and Kenyans who travelled a lot. But after a while, when they heard about the money eaten by KEMSA meant for COVID, for PPEs and other things, and it was heard that these people are ones the government is giving grants to from WHO and wherever else, many said it was now a disease of looking for money. Because, because, many said that if there is no money, figures go up, but when there is money, figures go back down. So, they are saying when the government wants to find money out there, they must keep the figures high. Now it was yesterday when Mutahi said that AstraZeneca vaccine isn't there, so people go to Johnson and Johnson. people said 'now you see this disease is all about money'. If there is no money… But if money comes, the disease goes down, but if there is no money, the disease goes up. And many ignore wearing masks, washing their hands, social distancing, because now even if you use public transport, you find the same passengers. There is no sitting far apart. there is no 'you sit there, you sit there', no. and if the numbers go down, they say covid is over. Money came and the disease is over. So, the community as per now see it as a disease of looking for money. So many say even if it's there, the government is looking for money with this disease. Yes.

Interviewer: what are the community perspectives on COVID about how it is spread? What does the community feel or think about the spread of COVID? You know, as community health volunteers, we have gone through training so we know but what is the community's perspective?

Respondent 1: many think this is a disease from abroad. But others say that it is a disease of the rich. The rich. They say that they are the ones who go abroad and they are the ones who take to those big countries. So, in that case us we can't get it. that's what the community members say. Because they say they haven't seen someone like the CC getting the disease. Many say that. Even the drunkards, you should hear what they say, the drunkards, they say they haven't seen anyone get sick with their eyes. That time they said they haven't seen it with their eyes. They just heard someone was sick with it. Just the other day is when they learnt that… but before when COVID just came, last year, second wave, people were saying it's for the rich. Yeah. That was their opinion. You couldn't get it; it was just for the rich. They said 'that one goes with big cars, that one was abroad, that one was [unintelligible].' But then during the third wave they saw that there isn't anyone who can't get it. Yes.

Interviewer: thank you very much.

Respondent 2: I think I-… they also said, this disease came from abroad. It's not for Africa. Because we are black, we can't get this disease. So, someone would say unless they are white, they won't be able to get the disease. Even if you told them that they can get it they would say no. They said this is a disease for white people the ones in America, China, the ones with white white skin. But as black people we can't get it.

Interviewer: so, what are the community perspectives on how to prevent COVID? Including the use of masks. What do the community think about this, the prevention of COVID? What are the community perspectives on how to prevent COVID-19?

Respondent 1: their opinion is that if you wash your hands, you are clean. Because we returned… There was sensitized. Even CHVs, there's none of us here who hasn't studied. And we were sent as gatekeepers in villages. We went to villages and said first of all, you wash your hands well and sanitize and wear a mask. The five -- we called it 5 rules, to wash what… to sanitize, we showed them. So, they believed if you wash your hands, wear a mask, keep social distance, you won't get COVID-19 quickly. And if you don't remove your mask. That's what the community thought. The community at large knew about COVID-19. They know. They know. Unless it's how our sister has said, ignorance, but everyone knows. On the TV - it's there, on the radio it's there. Posters are there. CHV's are on the ground. Everyone knows. They know that if you wear a mask well and you don't move it around you prevent it. If you wash your hands well with clean water and soap you will prevent it. And if you keep social distance. Yes. They know. They know.

Respondent 2: the community is funny. There are people who say the destiny of someone has already been ordained by God. If you are to die of COVID, it is already written. So, there's no preventing it. Yes. The ones who are to die of COVID will die, and the ones who will remain will remain.

Respondent 3: mine is about the lactating mothers. It's about the women who are still lactating, perhaps they have travelled around and they still have their small baby with them. They carry their sanitizer with them and they wear their masks, so you know they have understood well the concept of hygiene. When they walk, they carry their sanitizer. They wear a mask while they feed the babies. They don't touch touch things recklessly so that they maintain the safety of their child. Yeah.

Interviewer: **so, do you feel like the county was prepared for the COVID pandemic? do you feel like the county was prepared for the COVID pandemic?**

Respondent 1: I thought so, from the word go they didn't. yes.

Respondent 2: you know what I think? The county was not prepared from the word 'go'. Because, even to get the PPEs was a problem. Like me, myself, I was going to the hot spot areas. I was going to the hot spot areas, even during the fumigation I was going to the hotspot areas. And getting a PPE was a problem. So, the county was not prepared first, secondly, we didn't have a COVID centre. The COVID centre came later. Having a patient with COVID, to look for them a bed, it was a problem because I remember there was somebody who had COVID in my area and we found a bed in Karen. So that problem of finding a bed, finding a bed, it really messed us up. We had to…there was no other way to… then another thing, even when the county got the COVID centre, that is, I think Tigoni and Thika… the facility did not have all the required equipment's, so even to take a sick person there was a problem. So, it was even required that they go with an ambulance from their house. So, me, what I think, it was not very much well prepared. Yes.

Interviewer: **thank you. What did we feel, what did the rest of us feel, was the county prepared for the pandemic?**

Respondent 3: they were not prepared. Because… I work in Tigoni, the sides of CCC. And when we closed Tigoni, you know we closed all the departments? And we left and we went to Limuru Health Centre. That facility, did not have the requirements like Tigoni. There, the pregnant mothers couldn't have more than, maybe five deliveries. Or four. There is no ward to admit mothers there. There is no ward to admit male or female or paediatric, there is none. Now, they should have prepared first, saying we will close Tigoni first, then we go to a hospital which has things like X-ray, triage, ANC, maternity… but now they just suddenly closed it. We were just forgotten and confused. Because even the community was not

prepared. They were not prepared at all. Even that time, that whole year they were just struggling. They were just struggling. So, we would like that, if it's something like this that is an emergency, if it's something like covid, they should enlighten the community. Create awareness, so at least they are aware that we are doing this because of this. Then again, even now, we know that COVID is a disease that is preventable. and we have not received numbers like 200 even there at Tigoni, 200 people. So those beds could have been for covid people, we could have prevented it. Triage could have continued its work as usual. Maternity could have continued as usual, and we could have continued. But now we closed the entire hospital. The community was defeated, where would someone go? This question meant that if someone chose Tigoni as their hospital for NHIF, where would they go? And they had chosen that facility. These are some of the challenges people had. So, the county government should have done that somewhere else at least, so that people could at least… they could have viral there, NHIF there, facilities in one place. But now to close just suddenly people found it a challenge. They suffered a lot. But now we will return to normalcy.

Interviewer: **thank you so much for your information. Do you feel that the county is prepared for another pandemic, if it was to happen? You know covid is a pandemic. And there are many pandemics that have happened in the past. So, if we were to have another pandemic, in the country, or, you know a pandemic is global… it happens around the world. So, do you think that the county is prepared for another pandemic if it was to happen?**

Respondent 1: me, what I say is that, I don't think so. I think in the community people were preparing like a disaster has come. Or let's say the country as a whole. Since December they knew that there is COVID, and the first case came to Kenya in March so if they wanted to prevent it, they should have locked down in December so that all those planes…all those planes… all those planes… about the country being prepared for another I don't see they should have just learned from the COVID-19 that there is another disaster that can come. Another disease. So how are we prepared as a county? Let's say our Kiambu county. There was someone who talked about beds, are our beds enough? Are our facilities equipped? Because now there is a problem because we have been borrowing. Even the oxygen cylinders. You go and find that there is no oxygen and you have to go find it yourself and bring it for the patient. So, we should be asking ourselves as a county, are we ready for another disease which we pray does not come. But we are prepared. Let's say, here in Kiambu, do we have enough beds? Do we have enough oxygen cylinders? Do we have ICU beds? Do we have the capacity for the pandemic? And for the other patients? So, if at all we were to be

prepared we would be looking for other things. Let's say, the thing that caught us off guard. If it was the bed capacity, let's say we add about 50 or 100 beds in each facility and we increase some other facilities with more equipment to cater for any another pandemic to come. Yes.

Respondent 2: what I can add to that is that, for our county to be prepared, we must empower the community health volunteers. Because these people are in the community. They should be empowered with good information. Them. See they are the ones involved with polio, vitamin A, dewormers… its them. They are the ones who know every household and they should know every communication. They are level one. Empower the level one. Empower the level one. Community health volunteers. Be empowered. Even right now, we have told you about COVID, still we have challenges. For example, now you have to go see a sick person and it's raining and you don't have gumboots, you don't have an umbrella, you don't have a raincoat, sometimes you might not even have a mask, gloves… and then you are required to do what? You are required to go. But we just persevere and go. Even airtime. And even that person there, the ones who are home based, they aren't given airtime. You call with yours to ask how they are faring. And that airtime… God helps us a lot. We didn't have our own airtime to you know how someone is faring. Because you know, Because, you know, he is your neighbour. Your community member. And you know him. And we volunteer. First of all, county government empower the level one community health volunteers. That's the best thing. If you do that everything will be good, because information is done by them, sensitization is done by them, they are the gatekeepers. See its like that.

Interviewer: do you feel like the county is prepared for another pandemic if it was to happen?

Respondent 3: the county is prepared. Because even Tigoni, what I see, it has been developed. We have wards, we have beds about 300 beds. I think it's about three hundred. In the covid centre. But even if we have beds and some people come as asymptomatic, return to the community and the community people are here. Are they empowered? Are they empowered? Are they given airtime so we can talk from afar? now otherwise if they aren't empowered how will they talk. And even those who are home based they can talk through phones. Even I use phones to take talk to my clients using my phone. If I feel too long has passed then I request for a physical meeting even if I talk from afar, from the gate, we talk 'how is the family? how are you faring? any challenges?' so it may be good… yes, the county government they are prepared, on the other hand they are not.

Respondent 4: I hope they are not. In other words, they are, in other words they are not. Let me start that, because us, we are the CHV's working in the community. That level one should have now a bottle of sanitizer, mask and visiting the community - which we don't have. Then after that, they make sure that we have our NHIF cards ready. Because we don't have, others are expired, others we don't have… now we are told to go to the community to register the vulnerable within NHF cards but us we don't have period so please, look for us the machinery then the community will be OK. And then the county will be OK. Thank you.

Interviewer: thank you very much for your inputs. I would not want to close the discussion if you have a reaction or any other response to our question about the county preparedness for another pandemic if it was to happen. But if there are none, I would like to take this opportunity to thank you all for your input in this discussion, for this focus group discussion about the utilization of health services and COVID-19. So, thank you very much for your participation and for the good work you are doing in the community.

Interviewer: tell us your experience during the COVID lockdown.

Respondent 1: at the time of COVID I can say I have positive and negative issues that I passed through during that year. As a community volunteer we didn't have… we didn't go door-to-door to check for the clients that we had. We didn't have equipment that we should have used for that period. And still, the community that needed us didn't have any gathering to educate them in various ways. On the positive side as people were wearing the masks, we didn't have any diarrheal diseases, the people did not catch colds as per the research we have conducted as volunteers, though the problem was only on our side. We didn't do the jobs that we normally do the right way. The second thing that we had, I will take the side of disability that I work on, these children were not getting therapy. During that entire lockdown these children did not get therapy and when they finally did it, they had worsened. They have many issues including their health and the conditions they find themselves in.

Interviewer: did you say that…they could not access therapy services?

Respondent 1: yeah, they couldn't access. All the therapy places were closed during the COVID time. Now they are trying to open up. Yeah.

Interviewer: what are the challenges that you experienced as a community volunteer?

Respondent 1: the challenges that I went through… people were down and mostly the community volunteers that were in the villages take care of the needy and the highly vulnerable. They didn't have enough food. They were not given enough food by the government as they were promised to do. So, we had those issues in the community. And also, they couldn't access some medication maybe they have appointment from that county and they are sent to Kenyatta and you are refused to cross the roadblocks, it was another issue. If you had an accident and you had to go to the hospital you had to access that service through administrative police and sometimes, we were harassed so we had a hard time, we had fear. Many people suffered at that period.

Interviewer: anyone else would like to share their experience with covid?

Respondent 2: yes, at that time of COVID we also had a lot of fear. People were very scared of each other. It was said that the disease is severe, and people were told that we shouldn't be found together. So then when we used to go to visit clients, we had that fear. There was a time I went to visit my client who has HIV, I went to visit the client… but when she saw me, I wasn't in her house I was just in her compound just to find out how she's doing but I saw that she was very scared of me. But I did leave her

because she told me she was fine after I asked her how she was and that she is continuing to have her medications. And then the challenges that I had were there was a time I had a pregnant mother who was to give birth at Gachororo. At that time, it was required to call the chief for a letter because it was night time and then when he wrote the letter we had to pass during curfew and I had very many problems. The mother wanted to give birth on the way but I got her to the facility at Gachororo.

Respondent 3: as a community health volunteer, there are some challenges we have been facing during this time of covid. Like, most of the community members stigmatize the community health volunteers because most of the health workers had been infected with the disease so we when we went to the communities, we were told that 'you work in the hospital' and 'you work with health workers so you can get COVID and bring it to the rest of us. And then another challenge is that you would find that the number of home deliveries had greatly increased. Mothers give birth at home because they were scared of the hospital saying that 'if I go to hospital, I will get COVID'. And then there were places like Thika Level 5 if you tell someone to give birth there, they will say that people are kept too congested there so they are scared of the congestion in that hospital. And then you find that this mother does not have any money for a private hospital so she just decides to give birth at home. She just decides to give birth at home. Another thing during curfew many women needed to go to the hospital but this is on a positive note. There was a phone number that we were given as community health volunteers to sensitize the community members such as these pregnant mothers, that this number we told them to dial 9116 so an ambulance would go to them to pick them up so that they wouldn't give birth at home. Like on my side, on my side… like my area I used to call them and advise that at night you should call that number and the ambulance will come for you so that you come to the hospital and give birth here.

Interviewer: that's good.

Respondent 4: I am George. I have come from Gatundu. I am a community health volunteer from Gatundu South so when I used to go to the villages people thought that the government will just give us money and give us masks for free. We would say that this is soap, this is sanitizer, and they thought that we would bring it to them from the government but when we told them they had to buy it for themselves, there was a shortage of it at the shops, they would not find it at the shops. Even at the homes when we would say that you need to have a hand washing station outside your home, some of the people did not have it. Then people started making a lot of noise telling us that 'you are working for the government, there is a lot of corona, we hear about it on the radio and

see it in the media that the money is a lot, but now you are financially squeezing us. And we would explain and we would explain but the noise was a lot. And then when we used to go deep into the villages, to teach them about the preventative measures, some of the residents began to hide themselves. So, some of the old women that we would visit regardless of being community health volunteers they would hide themselves saying that we could bring them that disease. So, the challenges were a lot, we had a lot of challenges. When the churches locked down when the lockdown was just enforced, people used to stay at home and couldn't do anything. This has brought us a lot of issues. Even right now we are shocked when we hear that people are just dying you just hear 10 people died. So, you start to wonder who is speaking the truth. Members of the community think that these people are in the cities so the government has to have a will to differentiate the truth from lies. The profits have to be increased so that people **.

Respondent 5: OK as a community health volunteer, I have experienced many things to do with the community especially during this time of COVID. You would find someone who is sick at home, you find someone who is bedridden at home very sick, and if you have to mobilize people to take them to the hospital many people would just desert them. You, see? If you call the car the car that takes them to the hospital to take them everyone would desert them. There was a time that someone was sick at night and I was called. I had to call my superiors to ask them how to get an ambulance, but once you call for an ambulance it wasn't all the time that an ambulance would come. So, I had to apply myself fully to that particular job to make a way. So now here at level 5 they were not admitting. Many hospitals, to say the truth, were not admitting. So, we had to travel all the way to Murang'a because that is where there was no lockdown and there is where he was admitted. And because it was late, he died. So, we had that problem of people getting very sick at home and people would desert them and it was a very big challenge. Yeah. Also, if people knew that someone had a sick person at home, they would stigmatize that particular family. So, it was even difficult for that family to be checked up on because people were scared. And so, you can see that's bad. Yes.

Interviewer: thank you. Sorry about your experience.

Respondent 6: me as a community health volunteer there were very many challenges, I met last year. When COVID came to Kenya and the lockdowns were enforced no one was going anywhere no one could go anywhere and people were just at home. The clients that I was looking after who were on ARVs I had many problems with them. They would call me telling me

‘I have finished my medicine, and I don’t know how to get more medicine. what do I do?’

It forced me to buy them medicine out of pocket or contact my superiors to inform them that this client needs this medicine, they don’t have it so now what do we do? We would go around looking for these medicines but eventually we got them from the chemist to give the client. In fact, many of them did not have any food during that time of COVID because as a community they were economically strained no one was working. There was nothing you were removing from your farm. So that initial time when COVID hit there were a lot of problems and we had to pass through many challenges. There was a program that was started when COVID came, we were told we would give assistance to those who needed it the most from the government. But even till today those people have not been helped. And that program came in when COVID hit. So, we had a lot of challenges and we had a lot of problems. Even the list of names of the people who needed help there was no follow up on it. The government will know how it will help those people. So, then it brought in a lot of challenges, even today.

Respondent 7: as a community health volunteer, another challenge we face these mothers who were supposed to go for growth monitoring at the clinic, but those that only needed their weight checked were told not to come to the clinic. The ones that received immunizations were given, but if they were checking only the weight, they were told that there is no need to come to hospital and they could leave their children at home. So, you find that a lot of the children did not undergo growth monitoring. So, they would not go to the clinic and when we went there, we were told not to take children to get their weight checked so when we would go to the community, they would ask us if we would check the weights at the community level because the parents liked to see the weights of their children every month. Yes.

Interviewer: and were you able to do that? Were you able to assist them in terms of growth monitoring?

Respondent 7: like for me, there were some children we had. We had a BP machine and weighing machine so for these children we used to check their weight since we had the machine.

Interviewer: okay.

Respondent 7: however, for the issue of recording that is where we had a problem recording those weights in that book for the clinic because I didn’t have experience in doing that. Yes.

Interviewer: **okay. So, were you able to do your regular duties as a community health volunteer?**

Respondent 2: okay, the duties we do every single day as a community health volunteer were affected by COVID. In one way it was affected was that we did not have those protectives, we did not have masks, we did not have sanitizer. Since you know we are volunteers we did not have money in our pockets to go and buy those masks and buy those sanitizers so as a result even I was afraid because I could not go to a client just as I am because even the client themselves would be scared of me. So, we had a lot of challenges. you would find a sick baby… until we had a program that would assist us with those materials, those masks and sanitizers, which enabled us. But otherwise, you would just go with that fear even the client would be scared of you. If a child is sick for example we would go and measure that temperature. You had to somehow keep distance while measuring that child's temperature, and then you had to sanitize everything you used. Then the parents also had to sanitize. it was even better if you showed the mother how to check the temperature and they did it for you. Then after the mother checked the temperature you had to sanitize it again. It was hard work for parents to understand what you are saying, you see you are not close to them… but you see you have done something even if it not up to the standard we usually achieve. Maybe I can also add the issue of child clinics, many children had lagged behind on their immunizations. Some of them went late, some of them went private, because you see when they went to the clinic there was no work, there was nothing going on. So many of them were late and I know this because they were coming to me and we did not have immunization. So, I would just advise them where to go from my place, many of them opted to go private even if it was late. Yes.

Respondent 3: we did our duties. Okay… people were scared of going to the hospital. So, the responsibility fell on us to go and check on them, and maybe check on the children who have missed immunizations so that at least you convince them the risk because some of the facilities were operational but people were just scared of COVID. So, you go and convince them, the advantages of it or at least the reason that they should go and get immunized. Or if a person is sick at least they are not afraid so we had to do our work regardless of whether there was COVID or not, so we did it.

Interviewer: **anyone else would like to share whether their duties were affected, whether you were able to do your regular duties?**

Okay thank you.

So how did you overcome the challenges? The challenges that you experienced during the lockdown in terms of how you did your duties…how you were able to carry out your duties? How did you overcome the challenges?

Respondent 1: on my side, I deal with clients who have disabilities, and we have a support group. And so, I found a link from NGO's that would give food every two weeks and donors who would provide medications. I also joined up with other community health volunteers and we would go door to door. The government brought food three times and the distribution was done by the community health volunteers whereby we would take the food into the households. So, through that way we were able to overcome the challenges we had. And then on the other side of sanitizers and masks we were helped by the church, there is a Catholic church here on our side, so we would go to the church and they would give us sanitizers and masks. It was not much we were not able to reach everyone but we tried. The highly vulnerable people we helped them through this way.

Respondent 2: I was able to overcome my challenges because first of all I was filled with love for the people I serve. When I would visit them, I would ensure that they would wash their hands many times, wear their masks and also that they had sanitizers. At the facility at Gachororo, we would give them sanitizer. That sanitizer would go to a client that you saw had a lot of problems. So that is how I overcame my challenges.

Respondent 3: even me as a community health volunteer I would overcome my challenges the way we did it for our community. There was a time we went to a shopping centre and they did not have any tanks that we used to fill with water for hand washing purposes, nor did they have sanitizers. So, we had to contact someone at Kiambu to bring about three containers so that we could keep it… there were these young men here who used to eat khat who did not wear masks or use containers, so we placed it there and we told them to fill it with water and wash their hands regularly. Then there was another time we were walking in the villages and we found that there were some people who did not even have sanitizer, the old women and the old men did not have masks. So that forced us to go and find masks for them, we had a person who used to make the masks for us. As a community health volunteer when there was MAZIISHI we used to stand there at the gate we used to keep the container filled with water and sanitizer there. We had masks and we used to give it out to the people who were not wearing masks. And then when we used to work in our villages those women those old women who did not have masks, we used to work with them and give them masks. Even churches, even a few churches we visited and we

used to give out masks because there were many masks the person who used to supply them brought many masks. We used to show them these things… this information about COVID we used to tell them. All these things taken together solved our problems. All these things taken together solved our problems because there were some people who would sponsor these items. Thank you.

Respondent 4: as a community health volunteer, the way I overcame my challenges was that there was an organization in the sub-county called *Living Good.* It has helped us so much as community health workers in Thika sub-county. They used to supply us with soap every month, masks, cotton wool, they would give us even spirit so that we could go to the households and continue on with our work. For example, we would not lack masks. We were given boxes of 50 masks, they gave us gloves, so if I went to the community to do my work at least I was protected. So, they have helped us a lot so that we can continue with our work.

Respondent 5: what I can add is, we were educated about COVID so that we could go to the community and teach them about COVID. We were taught by the county by the subcounty, and then we were taught also by these donor empowerment… we had a few CFCs that would go an demonstrate to these people of the motorbikes you would show them how to wash their hands and sanitize, and teach them about COVID, and then we would take a picture that you would send to that empowerment group. This we are continuing even till today to continue educating them about wearing masks and the importance of washing their hands. Also, we would be provided containers from Mwenda*, we would get containers, not too many just about 55 that we would then take to families. And even that we used to go and check up on if they were being used. We would just educate them on how to prevent covid, but still the measures in place we are still trying to continue.

Interviewer: thank you for your responses. In your opinion, how did the community respond to the COVID-19 measures? In your opinion, how did the community respond to the COVID-19 measures? What do you think about how the community responded to the COVID-19 measures? These measures are hand washing to prevent infection from COVID-19 we have to wash our hands, sanitize, social distance… and even lockdown, see lockdown was one of the measures to prevent COVID-19? So, what do you think? What is your opinion towards how the community responded to the COVID-19 measures?

Respondent 1: I think the community in our area took it very seriously. Because when we told them every house has to have water and soap for hand washing, they adhered to that. Then, on the issues of mask you could see that at

least people had them. And then initially when it started people were not gathering. So, they were adhering to the measures.

Respondent 2: OK in the community we were able to sensitize them and people were able to understand the importance of wearing masks and washing their hands, especially the times when we were going to the households. We would teach them about the importance of keeping leaky tins so that everyone who would enter… even us we would act as a good example, when we entered a household we would wash our hands first, to show them what was required. They were following our instructions. They were also empowered with a lot of information because everywhere you looked the information was being shared. So according to me they followed our advice well and that is how we prevented the disease. Yes.

Respondent 3: the community's response to COVID and these measures against COVID the first time it came people took it seriously because it was a foreign disease and people did not understand it. So, they had a lot of fear and all the measures that they were told to take they took them seriously and adhered to them. But after the lockdown and then reopening and then wave two people stopped adhering to the measures, they said that this is a government project and that the government is looking for money. Because they reopened and people traveled, people went everywhere so these measures during round two… wave two were not taken seriously like wave one when it initially came.

Respondent 4: when the community heard about COVID people lived peacefully. Even the old people would live at home with everyone peacefully. You would go into a homestead and water was kept nicely at the gate and you would wash your hands first before getting close to the family and even as you approached the family, they would tell you to sanitize. Even if you carried your own sanitizer for example as a community health volunteer, I had my own but it was like mandatory for them to offer their own sanitizer before you begin talking to them and you also kept social distance as you talked to them.

Respondent 5: what I can add is that it affected us a lot in our area Githurai where by these young girls at home would get pregnant and they would be doing their exams while they were pregnant. so, this was the challenge that we had. And the DOA and DCC took those names but we don't know the reasoning behind doing that or how that has helped the girls. This is the negative response or the negative experience I had because of COVID.

Respondent 6: at that point things are going very badly because…of the reason of masks. when the government said wear masks people did not want to wear masks. When they were told to keep distance, you could see that they would stand close to each other. We were very disturbed you felt

defeated when you would tell them to keep distance but when you reach there, they are close to each other. Okay, during the time of lockdown…lock down like for example us old men we used to be disturbed a lot because we were not able to reach home early enough. you would stay behind and talk to your friend, you were selling your plot, you are doing other things, but now when we were locked down you were just sitting at home. Even if you wanted to sell something there was no way you would sell it at that time. During the time of lock down there wasn't even a single time when people had fun at home. The wife would just insult everything and you did not have any money. They were just fights there was no money. You could not go to work. You could not go anywhere. You could not go to your friends. You would just get angry all the time. We had a lot of challenges. And even when you go to church, the government said that people shouldn't go to church in large numbers [unintelligible]. Some churches… there are those who belong to certain churches/own certain churches… some churches are not made of stone and have been eaten up by termites. There are some people who volunteered to wash the church and others weren't going to church. That was some bad stuff we are praying to God for it to end. Thank you.

Respondent 7: what I can add is that during the time of COVID people took it seriously. The bars were closed and there were not many drunkards. So, then the young men would be at home and would do their work. Because that time before COVID they would just go to the bars and you would see many young men were drunk in the streets. But during lockdown and during COVID young men calmed down and helped their parents at work. And the community took it seriously. Because we had a good time as the sons helped their mothers with all the work. So, when COVID broke some things down it also built some things up because these young men were drunk. That's it.

Respondent 8: to add to that on how the community responded to the COVID-19 measures, the business premises had to have a hand washing stations so that if anyone came to buy anything they had to wash their hands first. But you notice that many of the businesses did not have these hand washing stations saying that they could not afford it. Then also for masks, many people said that they could not afford the masks so the government should bring them masks. But majority started businesses for selling masks and that many of them began to sew masks. So, some people found jobs sewing masks and other people came to buy them.

Respondent 9: so additional for that is to say first of all the time COVID was publicized the whole county was afraid especially in our area the main point of interest was that all the people began to run home. COVID brought

some unity to some households that wasn't there before. It brought togetherness in our country and new ways of washing, washing hands, to wear masks, right now there are some illnesses that disappeared without us knowing but we were measuring COVID. COVID has taught us very many things e.g., for there to be peace there must first be togetherness. Two, there are some other issues you see getting publicized that are not in the department that we are in e.g., there are those people who say they are 'sex workers' whose business went down since every man was following their wife to stay with them or to stay with their family. So, these 'sex workers' became desperate and they were our clients as we are the CHVs and we were working hard to know their conditions and how they were faring. When they were affected, we got so many complaints like 'we don't have flour, we don't have accommodation, we don't have money for rent', and this COVID still has added more issues because landlords got big challenges because people did not have ways to pay rent. Yes, they were renting that house and you know now they were staying without paying rent. We saw that COVID brought many issues, yes, it's a big challenge for our country because those with extra-marital affairs it's better that people stay the way the bible says as one. Thank you.

Respondent 10: it reached a point politicians resumed political rallies so people, the community were relying on the leaders by watching how they operate. So, people saw the political rallies are ongoing, so this stopped adhering to the measures. People began to go to meetings like the meetings for dowry those resumed. Because they saw the politicians are still going for rallies.

Interviewer: thank you. Okay so in your opinion how did the covid-19 control measures affect the community access to health facilities? In your opinion, how did those measures that we have discussed about, lock down, hand washing, sanitizing, social distancing… how did those measures affect the community access to health facilities? The people in the community, how were they affected in terms of accessing the dispensaries, the health centres, the hospitals?

Respondent 1: first of all, the time COVID just began, I think the health workers were scared of covid. They saw the members of the community with the ones bringing covid. So, you see they would tell them 'Stay at the gate' then it was like they were preventing themselves from getting coronavirus so they didn't mind. The community saw that since we are the ones taking corona to them let's just stay at home. So, people kept off from the health facilities.

Respondent 2: okay to add to that I can say that most of the community members found that since there was cessation of movement most transport or

public means were not available to them. So, it was required to take a taxi or a motorbike and then also the fare was hiked. Then you get to the roadblock and you asked where you are going and you remove a paper from the hospital and say I am going to hospital. So, to avoid all that you just stay at home. Assuming your HIV positive patient and you have your yellow card and you're stopped at the roadblock so every time you go to show your yellow card, every time you go to show your yellow card… so you find that even the stigma increases so people saw it better to stay at home rather than to reach the police checkpoint and remove their yellow card saying that they're going to get their medicines for HIV from whichever dispensary so most people would opt instead of paying for all that transport reaching the roadblocks and then removing their cards to just stay at home.

Respondent 3: At that time, we had a lot of problems. Because curfew was enforced, even motorbikes didn't go anywhere, cars were not on the road. to have an illness meant you had a lot of problems and not all of the people knew that 1116 number. Many people died because they didn't get access to doctors. they just died.

Interviewer: thank you. In your opinion, how did covid-19 control measures affect the community capability to deal with other priority diseases? How did those measures affect the capability of the community to deal with other priority diseases? Other priority diseases are diarrheal diseases, diarrhoea, you know? Cough. TB. Malaria. Wounds. So how did those measures that were imposed on us, because its all of us, how did they affect the community capability to deal with other priority diseases?

Respondent 1: on the positive side, because of consistent hand washing and wearing of masks there was no dust or dirt in the mouth. With hand washing there was no diarrhoea. Even TB that infection of the virus in the air was prevented by the mask. So, on the positive side that was that. But on the negative side people were at home so someone with diabetes didn't get the required meals and that was compounded by not having medicines. They couldn't go for their medicines and that was the negative effects that affected us. That's what I have.

Respondent 2: these COVID measures the way they affected the community to deal with the priority diseases let's say for example the diarrheal diseases, the way my colleague has said on the positive side, the diarrhoea cases went down. Because people were washing their hands even the children were taught how to wash their hands. But the problem was water. You can find like Thika sub-county water was rationed. So, you get some places getting water only twice or thrice a week, so people did not have access to water all the time. People would ask you 'how do I wash my hands when City Council has closed off the water supply?'. So, they

didn't get water. Those illnesses like diabetes, blood pressure find you find someone without money to buy medicine because maybe for example they had a business… I had one case of a woman who would fry *mandazi* and she is a hypertensive. So, this woman her sales went down and she had no money to buy medicines so it was required for me to connect her to an organization called *Macheo* for them to help her, support her, buy her medicines. Yes.

Interviewer: Thank you for that. Anyone else would like to add about how the community was able to deal with the other priority diseases?

Okay.

So, what will the community perspectives about COVID, its origins, how it is spread, how to prevent it… including the use of masks. So, what were the perspectives of the people living in the communities about where it came from, that's the origin. how it is spread, the way it would spread. and how to prevent it. So, what do the people say, from the communities that you come from about where COVID came from or what is this that brings about COVID.

Respondent 1: in our community when it started, they said that it was created by China because they wanted to make money so that they sell masks, oxygen, yeah things like that. But after while people changed. They saw that this thing is serious. Yeah. That's what I have.

Respondent 2: in the beginning COVID was something that was not well understood among the people because it was like something that just came out of the air. Everyone was scared of it because we didn't even know the symptoms. If you had a husband, you were scared because if he went somewhere, you were thinking that he will come home with it. So, in the community people were scared if they saw their friends, they would get scared. But people have gotten used to it they've started to meet each other and like they were told by the CHVs about how the disease is spread so they hear it on the radio and now people are not so afraid. But they've reduced practices such as hand washing the way the government has asked them because they are now used to it. And they don't find it too different.

Interviewer: thank you.

Respondent 3: about the community perspective about the origin of COVID, many said that it was a disease from China for Chinese and it can't kill Kenyans because Kenyans eat food that gives a lot of strength and Chinese eat food that does not give them strength. Our bodies have what? Strength. So, there's no way we can die from it

Interviewer: **what did you say about… what was the community saying about how this disease is spread?**

Respondent 1: OK, about how the disease is spread in our area people would say that it spread through sneezing, touching surfaces. If someone has a grudge against you or wants to harm you, they'll infect you by touching your things so that you get infected.

Respondent 2: in our community, they said that it came from China. But they did get angry during wave two because they came from airports and they didn't know where they went. They said that the government played a part in it so that Kenyans would die. People heard that bodies were just being picked up in the capital city. But here people were not dying like that. They said it was because they weren't eating well, they ate things from the fridge while here we were eating fresh food. In the bars, they would say they would say that you are drinking alcohol but you are also washing your hands with sanitizer, so you won't get COVID. They don't believe otherwise. Many drunkards don't wear the masks and if they do maybe it's just to avoid getting arrested by the police. Another side to it is that, the time it came here they believed it was a way for the government to make money. So, they didn't care. Maybe only because of the police they complied with some of the measures.

Respondent 3: the few things that I can add are that when COVID came people were told to wear masks. People were told that the mask is yours, you cannot use your friend's mask. So, these people thought, even the people who are making the masks that they all want money from us. Initially they thought that the government would provide the masks but eventually they were told to buy the masks for themselves. So, you would be shocked that you had to buy a mask for yourself every day. Initially we were told to buy one mask per day and then you would throw it. The next day you would buy another mask. So now you were stuck, where would all this money come from? If yesterday I went and bought a mask and now today, I'm wearing another mask and it breaks… you see it's good business for those people who are making the masks. Because today you wear a mask and it breaks, tomorrow you wear a mask and it breaks, and then you know there were some people who are going to collect these masks that have fallen on the floor, they wash them and then sell them to us again. Do you see it? You even get allergies. I wore another one in the morning and I started to cough I just threw it there at Kenyatta Road because I bought it from town. So now, if you took that kind of a mask to your grandmother in the village, won't she say that it's you who gave her that illness? It's like Uhuru said, people should take note and wear masks. And keep social distance. Even that metal rod to enter the buses you were told to stop touching it because that's how

you get sick. There was a time I saw a woman almost fall down because she did not want to touch that metal rod to enter the bus out of fear. She would enter the bus like this at the bus station… she was even grabbed by the conductor because she was standing like this. she didn't want to hold the metal rod. She was saying that the metal rod is the one giving her COVID. So, we were at a loss of what to do about this situation.

Respondent 4: I want to add something about how this disease is spread about how people would hawk masks. It was like every single person entered the business of selling masks. And for example, these people who were sowing the masks I would see the customers try it on themselves to see if it was fitting them properly, and then return it to the person who was selling the masks. If you tried to confront the hawker, they would tell you that they are conducting their business and you should conduct your own yourself. So, this issue of hawking the masks everything should have been restricted in my opinion. At least have a designated place where people could safely get the masks. Because everybody is selling, and you don't know if this person has COVID or not, and people were touching touching, even those ones who sowed the masks. So, this should be restricted so the disease does not spread too much.

Interviewer: **that's interesting. Thank you so much for your input. Anyone else would like to comment or to give their input about what the community thinks about the origin of COVID?**

Respondent 1: what I can add to everything that has been said is that… everything that we are doing, I hope, I don't think there is a single person who doesn't understand what we are doing… but there are still people who say that this is a project. The way they make the connection is that, for example, the times we would hear that Al Shabaab has attacked a place and when COVID came and we began to take precautions diligently, there was no person who worked like us CHVs did explaining to people about COVID. They would say this is a government project to look for money from us, for example, ever since covid came, have you heard about Al Shabaab attacking any other place? There has not been any. These are the things that they are spreading around… for example, especially us men, we have our different ways of looking at things. The way you look at a situation is not the way I'm going to look at the situation. And your opinion cannot be the same as mine that's what many of them were saying. Project! It is among all the other government projects that are purposed to get the government more money. E.g., when masks were being advertised, just the way my colleague has said, masks were now the biggest business opportunity. Since all the other businesses were closed people rushed to start making masks. There was no one, even

those ones who are selling vegetables, now they were selling masks. Go to the kiosks, masks were there. Go to any shop, there was no shop that was not selling masks, masks were there. You could not fail to get a mask. So, it shows that, I don't know if I should say this is the complete truth or a lie, we as CHVs, we have gotten these comments that this is a project like all the rest. We were told to close our mouths and other people were eating all the money. E.g., there was this foundation for cancer, and there were these sagas where they showed that people stole millions, billions and us we are here on the ground being told to shut our mouths and keep quiet so that you don't do what?... for example, here on the ground the way we believed it is that we couldn't say that COVID did not exist. Because I witnessed many guys who were infected by it. And they were really affected by it. But yet again people started to say that this is an illness of the rich people. These were the classifications of the disease given by the people on the ground. They were really confused. When we would talk about covid, you would see in the media that NHIF would not assist those who were infected with COVID. It was like there was some sort of discrimination going on. Everyone had to chip in for the funds, but when it came time to use the money for the people who were affected, they could not give them the money. The other day, people were asking me, listen, we put this money in NHIF, why can't it be used to help someone who has COVID? What is it for then? And it's like all these other diseases have been finished off by COVID. For example, ever since COVID hit, every single day we hear in the media about how many people have been infected with COVID. Did COVID come and eat diabetes? Did it eat TB? Did it eat Malaria? I can witness that diarrhoea ended because of people washing hands and maintaining hygiene. COVID has advantages and disadvantages. Thank you.

Interviewer: **thank you. Is there any other input about where the community thought COVID came from and how it is spread?**

Respondent 1: for example, in the villages people did not believe it was a disease that could affect them. It was a disease of the city. It could not reach here [the villages]. Unless someone had it…it was like it would only affect people of Nairobi, Thika, and those other towns. But, even now, people are not scared like how you go up-town and see how people are serious in prevention. So those people at home are not serious, not very serious in prevention because not many believe that this illness can come to them at home. Yes.

Respondent 2: I can add to that. It is true here at home they say here there is no COVID. And they say that… I can add to what she said. Me, there is a time I was visiting my home and I used a motorbike… in the car I was wearing a

mask and there was a preacher there preaching… and many people were seated there and not a single one was wearing a mask. So, when I alighted from the vehicle, the pastor stopped what he was preaching and said 'People have come here covered up and this is an embarrassment to God because only the chosen few are going to get sick. So, there is no COVID'. He told people to repent and ask for forgiveness and if they do so, there is no way they will get infected with COVID. So, I just left slowly with my mask on and left him preaching there.

Respondent 3: what I can say is that, those families that have had someone infected by COVID have taken it so serious. But those people who have not had a family member be infected with the virus do not take it so seriously. So, they don't have a stake in it. The ones who have had a family member affected, who got sick from it, they are trying so hard so hard to prevent it. They take is so serious. But the ones who have not had anyone affected they resist; they don't have a stake in it they take things easy. Yes.

Interviewer: **the ones that took it seriously, how are they preventing themselves…**

Respondent 3: they are not.

Interviewer: **you had said people who had been affected by it took it seriously while others who were not affected by it didn't take it seriously. How exactly are they taking it seriously?**

Respondent 3: yes. Usually have a tank of water and soap outside their home, so you can't enter their home without washing your hands first. And once you enter their home, you typically find that they have sanitizer. But those who have not had a family member be affected, they don't even take part in washing their hands. Yes.

Respondent 4: in our community, right now is when they have started being good. In many dwellings we have tanks of water outside the homes. They have taken that initiative of washing their hands. Many of them, about 70% have responded well in hand washing. The ones that are ignorant are the ones who drink alcohol and eat *khat*. Those people don't take initiative. But if there's a mother and her children at home you can be sure that they know about hand washing.

Respondent 5: what I can add to that is in many families, there are those that use these medicines of… maybe they buy lemons that they boil, they drink and they even give it to their children. That is what they use together with hand washing. In many families, you can't miss lemons and those families usually have someone who was sick. They also use herbs and

barks from a tree and many of them boil it, cover themselves with a blanket and sweat. That's what they do nowadays. Thank you.

Interviewer: **thank you. Do you think that the county was prepared for the COVID pandemic?**

Respondent 1: no, I don't think the county was prepared. Because even that time when the counties had to become independent and look for ICU beds you would find that especially Kiambu, you would hear that Tigoni has isolation units with ICU beds. But when you had a case of someone being sick and they were referred from let's say here Thika to Tigoni you would find that they were returned to Thika. So, we were not sure if they really had those ICU beds or if the situation was that they were at capacity. If the case was that they did not have those ICU beds that would be so bad. And if the case was that they were at capacity it would mean that the county underestimated how COVID would affect the people here because I believe our hospitals should be able to handle all the patients that come. So, no, the county was not prepared.

Respondent 2: the county of Kiambu was not prepared because when people started getting infected, they said that they would pay their own bills. The people had many problems. Most of them were isolated in their own homes for 14 days and they would emerge only when they were fine.

Respondent 3: I think the county was not prepared because you find that, like masks, if they were prepared, they would have been able to supply masks to the community. That way everyone would be able to wear a mask. And then, the CHV's on the ground should have been on the front line. Just when COVID hit, if the county was prepared, we should have been called first because it is us who create awareness in the community. We have an existing rapport with the community such that the information we give to the community members is taken seriously. So, the county, the first thing it should have done was to call all the CHVs. They should have been taught well in the first place so that they could go and teach the members of the community so that they could take those measures, but they did not do this immediately. They came to call us CHVs later on. Thank you.

Respondent 4: what I can add is that, it is true the county was not aware. Even right now is when they are rushing to build those hospitals, which is very encouraging and they are doing good work. But they don't see that, if they had alerted themselves right now, they would know… there would be… what's it called? The outbreak of these masks? We closed those issues with papers. Right now, if we are not cautious with masks they will come and block along with other things we are doing. Because if you go to see the waste, most of the waste is what? Masks. And that is where

you get those people who wash those masks, dry them, and in ways you don't know… it is ironed and returned to the market place. So, what I would say is that, for sure the county was not prepared enough to fight COVID. COVID measures should be enforced and our… okay… we volunteered there in the villages as per usual because we are the first guys to know that…to place that stress of any disease. We are the ground… what is it called? Zero level. We are the ones who are on the ground. We have been trying to explain CHVs are people who volunteer. Which is the truth. But us CHVs there is no one who has remembered us. For example, the county itself, has not recognized the work us CHVs have done. The county itself has not even asked 'who are the people on the ground?' but …for example, there was a time there was a strike… a small example, CHVs, whoever introduced themselves as a CHV, they can tell you how they have met so many challenges because people come to you regardless of whether you are a doctor because you can help them get to the facility they want to go to. When COVID came and it was evident that, in my area, one week, I recall in my mind very well, I volunteered myself to go and help whoever … because someone will have gone to KU referral, Ruiru, and they were told to go back home. So, it was a challenge because even me myself I was not taught. It just comes with other information and you read whatever you can. So, I was not taught how to handle the COVID-19, and you find yourself in the line of work whereby even you yourself don't know what to do. Yes, we have volunteered to do this work. But the county should remember us too. We can have some courage to do what needs to be done for those we serve. So, it's true. I would like to encourage this, the O level CHVs, are level one yes, level one, should be remembered as their own group. Because those others, level four level five… there was a time we heard they were on strike and those challenges came down to us CHVs. So, we have this burden and we kindly request the government remember what we did during the fight against COVID-19. Thank you.

Interviewer: **thank you. Do you think that the county government was prepared for the COVID pandemic?**

Respondent 1: at the start, I can call it, they were a bit prepared. You can give them a score of about 30%. Why? We as CHVs we are trained. We are called several times; you are called there at the sub-county and we are taught issues like we were taught about covid. So, we know things about covid. We continued to attend… there were a few of us who were chosen and we were given the first aid kit, the one we carry which contains medicines and everything you need. It has those things, those medicines we are given, we took them and things went on as usual. So, these were the regular on-going things. But the problem is here, the reason its 30% is that, we went and we were taught but we were given one mask. We

were not given sanitizer. I remember in our sub-counties… to start teaching the community members now there was nothing that was protecting you as you work. So, they can improve in that every time they educate us and send us to the ground, we are ready to work, we are ready to do anything they want us to do, we are ready to take care of the community, we have the skills, we have volunteered, so then when its COVID or other diseases that come they should give us protective equipment that is enough for us to work on the ground. They did 30% which is not good.

Interviewer: **thank you. Do you think the county is prepared for another pandemic if it was to happen? Do you feel the county is prepared for another pandemic if it was to happen?**

Respondent 1: with COVID I don't see it like they are ready. Because we are hearing, see it will come in July? We are hearing the rise will come in July, the one that is coming from India. It has already reached Kisumu. And if it has reached Kisumu, see it will reach here soon? And yet there is nothing we have been taught and there are no skills that we have gained. And we hear that one is very bad; people are dropping dead unlike anything before. Now what do we have as community health volunteers? We don't have anything. And in my opinion, I don't see it like they are ready for it. In my hospital like in Ruiru, there are no beds and there are no chairs. There is nothing, there is just a hall that has been built. On my side, they are not prepared.

Interviewer: **OK, just to maybe make you understand or elaborate, we are still in the COVID pandemic, the pandemic is still here with us. We are still dealing with it as a nation, as a county. There are many pandemics. There have been other pandemics previously affecting the entire world so if we were to have another pandemic, apart from COVID, do you think the county is prepared?**

Respondent 2: for me, the answer is no. they are not ready. Because if another one comes, another one that is worse than this one, that is the end of us. Because first of all, my reason for saying that is, it is true that we don't have the essentials or the gear to show we are ready. Those things are just like 'show-off'. Those things come, we take pictures with them, and then we put them aside. For example, when COVID hit for real, it was here in Kiambu, the first sub-county that was attacked was Ruiru. Because we were there neighbouring … so when COVID came, and I remember in my mind well, I recall my mind very well, because I remember people had gone to the doctor and the doctor did not know how they could handle those patients. They didn't have… what's it called? The education. They didn't know. It was a foreign thing. If it

comes again, the way I see it, only God can help us. My answer is no. they are not ready.

Respondent 3: I can say no. The county is not prepared in terms of… like now, they would be thinking about what funds to give people to cushion themselves against the COVID-19 impact. But right now, people are still dealing with COVID, they are dealing with other issues, God forbid another pandemic should come, I don't wish to say that we will be finished like someone else has said, but to be honest, we will be in fear. In case something happens, we won't know where to go. Because sometimes we think that the county government does not look at these things seriously. I remember we had a meeting with some people this week and they began to say that 'why are children going to school without a mask?' or the masks are torn, as in, if you even go to those schools there are no tanks for water. Of course, we know that children don't know how to use sanitizers. So, if the school itself does not have water for their children to wash their hands with, the children don't have masks, maybe maybe it's a big burden for the parents to give the children a new mask every day, so the ones the child is using has begun to tear, and yet the county, or the government as a whole, is doing nothing about it. So, if we are not able to deal with COVID as of now, what would happen if another pandemic was to happen? I'd say no we are not prepared the county is not prepared for anything more.

Respondent 4: to add to that I will say they are not prepared even according to me. Because, for example, that hall see it has been built and it is ready? So, if we were to face another pandemic or if another emerges, we can use those houses. But it's not enough. I will give them 10% because they have tried, and they must stay vigilant to see what can emerge they are ready. Those people in the offices won't listen to what's happening on the ground. For the people on the ground, we have everything for them, but those people in the offices know nothing about the area, nothing about the community. So, when they give their results, they give something narrow. So, it goes back to us down there, we give them the results and they should plan with us so that we can assist.

Respondent 5: my view is different, for me I see like the county is a bit prepared. For what reason? This pandemic hit us and we can say that we've been burnt by the fire if we have been touched by the fire. So next time you will be afraid of the fire. So, this time we had been hit by the pandemic of COVID and next time at least they will be prepared because anything can happen. So, they are prepared, but not a lot. So, if another pandemic hits, there is a way we can fight it because we have been touched by this one.

Interviewer: how do you think they are prepared?

Respondent 5: OK. I suppose they will try to find funds to keep aside for help in case of another pandemic.

Interviewer: okay, thank you. Any more inputs that you may be having towards the preparation of the county in case of another pandemic?

Respondent 6: what I can add to that is that these people have already prepared. Like us, community volunteers from Gatundu South, we have been given the first dose. I have my message right here, I am waiting for the next one in June or July, so when you go to visit the ground, you must, as a committee member, as a school-goer, you must as a community health volunteer receive the first dose, the second dose, the third dose, because you are the one visiting the homesteads. I would say they are prepared but they are going the way the country is going. Okay.

Respondent 7: I would say that they are not prepared. But the problem is the ministry. Those people high up, when they are given resources to give us people at the bottom, they just get lost up there. So, if you go to a facility, you find that there are things you won't get in the facility. In the isolation rooms you find that there are no beds, there is no that air… but the money is there. The problem is up there unless the government intervenes and make sure the money reaches the grassroots of our facilities. Then is when they will be prepared to help the community.

Respondent 8: what I can add to that is to say that, for example, our area Gatundu North, it would be good if a hospital like Ikegania can be upgraded somehow. Then if we had such a thing, the people within the Gatundu sub-county can be treated there and receive all their medications there. That's it.

Interviewer: thank you very much for your inputs. Thank you very much.
