# Supplementary material for: Community health volunteers’ experiences during the COVID-19 pandemic in Kiambu county, Kenya: A qualitative study
Source: PLoS One. 2025 May 7;20(5):e0322642. doi: 10.1371/journal.pone.0322642 (PMC12057936; doi:10.1371/journal.pone.0322642)
Supplement: S2 File — Contains the desk review on the state of the community health level. (PDF) [file pone.0322642.s002.pdf]

The level 1 desk work results.

Introduction

The following figure show the trend of different parameters for the level1 health services for the three years – 2018, 2019, and 2020. The 2018 and 2019 years were used as a control to show how services would have been in the absence of disruptions brought about by COVID-19 pandemic. Figure 1 shows the number of CHUs that were issued with any type of commodities comparing between the year 2020 and both 2018 and 2019. The numbers started high in 2020 January and February before reducing in March to July 2020. There was a spike in August and September 2020 after which the numbers went down to 0 from October to December 2020.

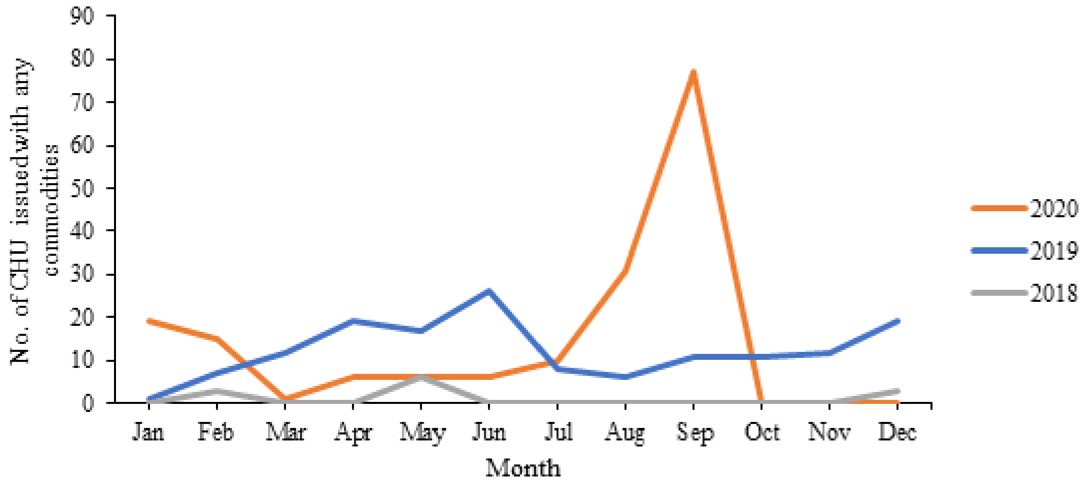

Figure 1: Number of CHU issued with any commodities

Figure 2 shows the total number of community actions days by the community health workers. These are the number of days that the CHWs were engaged in each month.

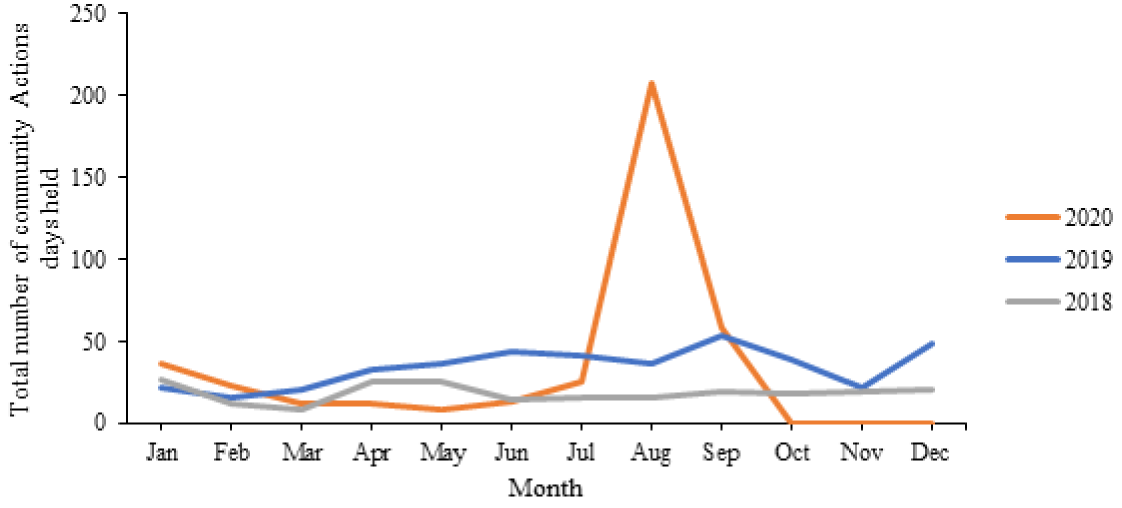

Figure 2: Total number of community action days

Figure 3 shows the total number of community dialogue days that were held by the CHEW. The trend in 2020 was similar to those in 2018 and 2019 for some of the months except for March and October to December 2020 when the numbers were lowest.

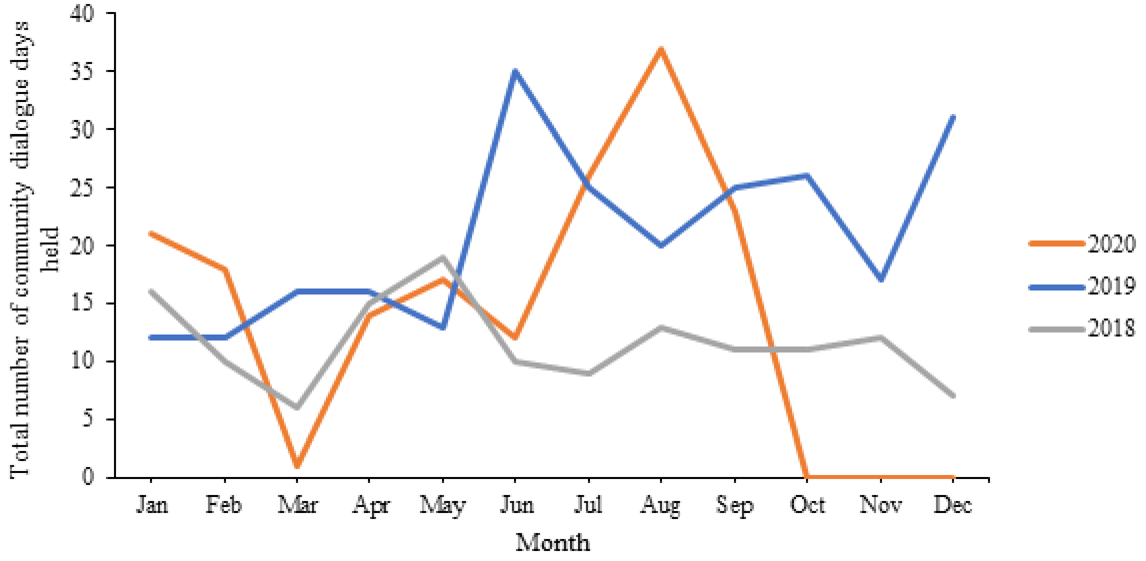

Figure 3: Total number of community dialogue days held by the CHEW

Health Indicators

Several health indicators were selected and their trends in 2020 compared with the trends in 2018 and 2019. Figure 4 shows the number of children aged between 12 and 59 months of a get who were dewormed.

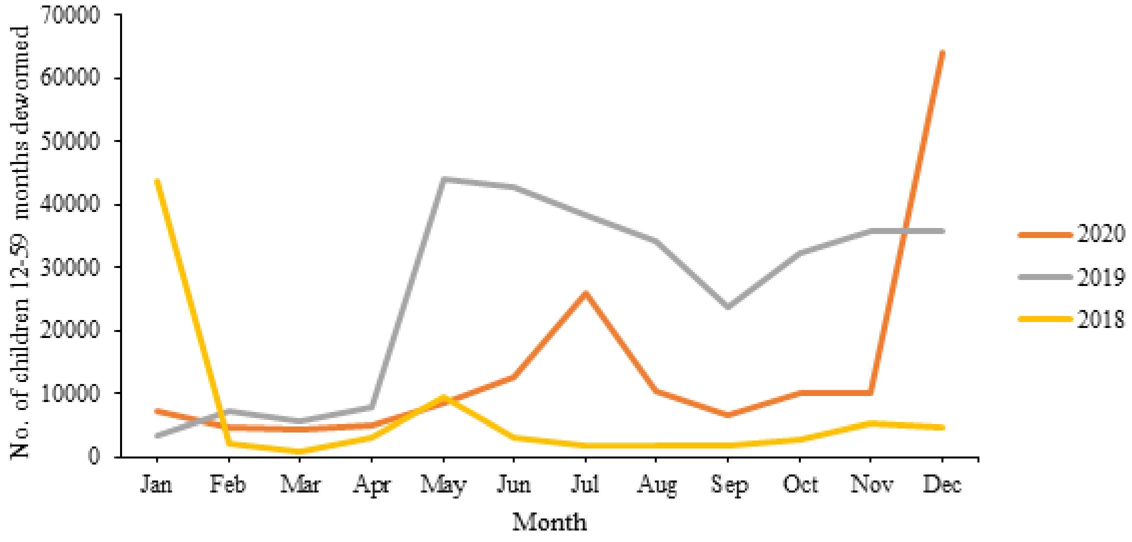

Figure 4: No. of children 12-59 months dewormed

The numbers of new-born babies visited within 48 hours of delivery in the three years are shown in Figure 5 below. Unlike in the other two years, the numbers in the year 2020 had two peaks one in February and another one in July.

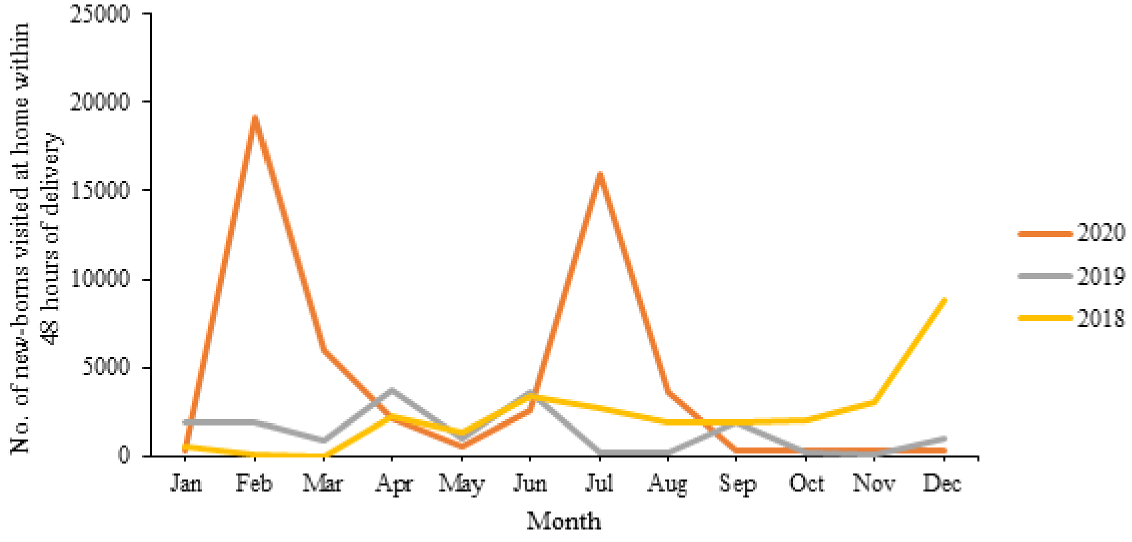

Figure 5 No. of new-born visited at home within 48 hours of delivery

Figure 6 shows the number of pregnant women who referred to health facilities by the community health workers. The numbers in 2020 were slighlty higher from May to December compared with other two years.

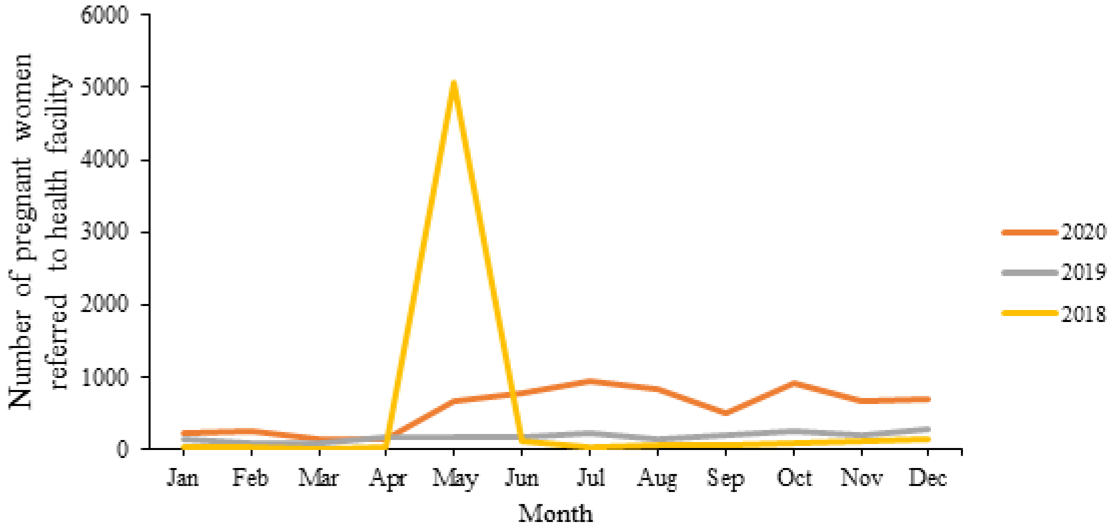

Figure 6: Number of pregnant women referred to health facility.

In 2020, the number of persons aged 60 years and above who were referred for comprehensive geriatric services for checkups increased after April compared with same months in 2018 and 2019 (Figure 7).

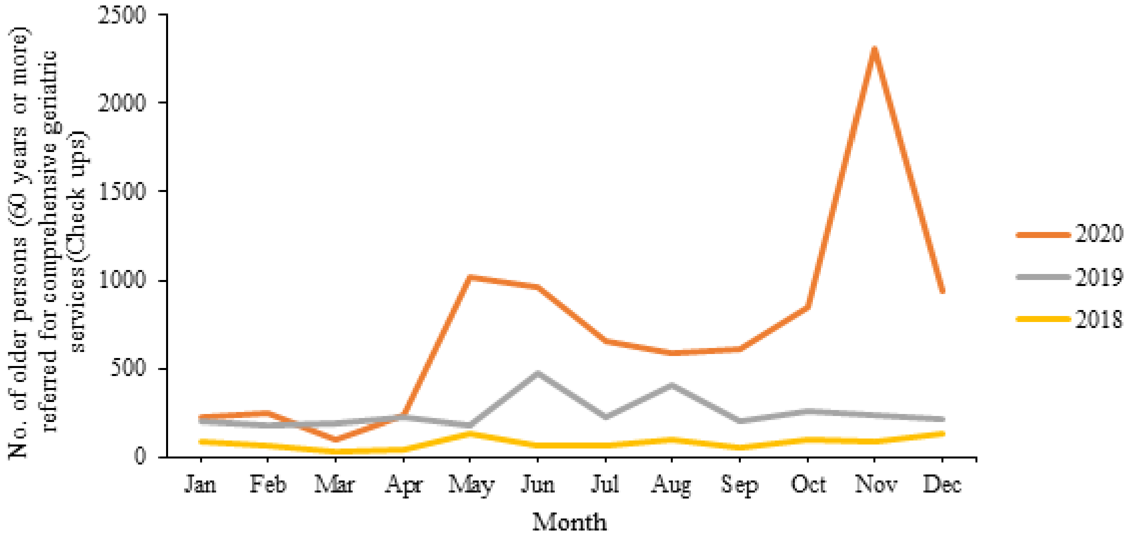

Figure 7: No. of older persons (60 years or more) referred for comprehensive geriatric services for checkups.

The number of children aged 12-59 months with zinc and oral rehydration solution (ORS) reduced in the year 2020 compared with the other two years from the month of March 2020 with the months of October to December recording zero children (Figure 8).

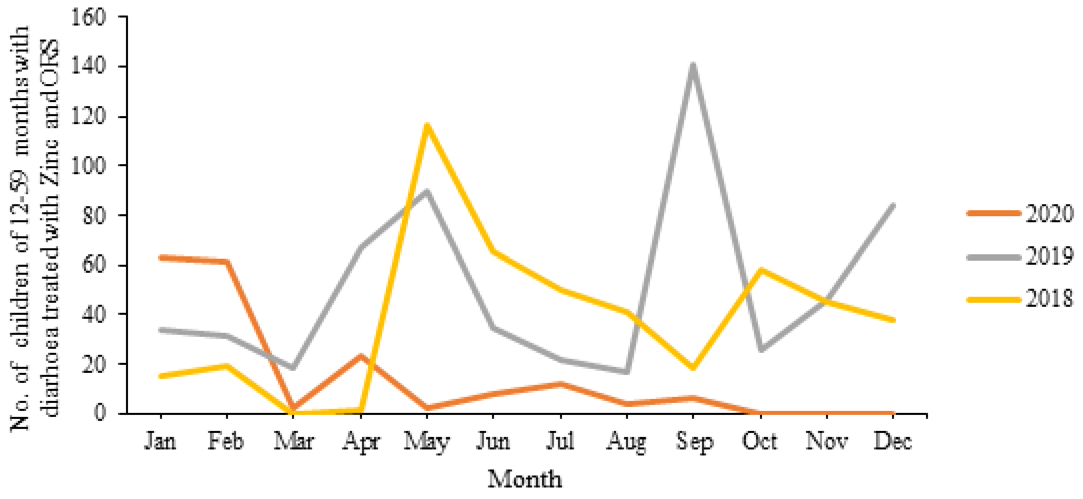

Figure 8: Number of children of 12-59 months with diarrhea treated with Zinc and ORS.

The number of referrals with cough for two or more weeks started high in 2020 but the numbers reduced drastically from March to May 2020. The numbers stayed lower than the previous years until September when there was a spike before dropping to zero from October to December 2020.

Figure 9: Number of referrals with cough for two or more weeks

Fever is one of the major symptoms associated with COVID-19 disease. The number of fever cases that were managed in 2020 reduced from the month of March with no spike May as was observed in May 2018 and 2019 (Figure 10).

Figure 10: Number of fever cases managed

Unlike in the previous years, the number of children aged 6-59 months who were referred for vitamin A supplementation started higher in 2020 than in 2018 and 2019 before experiencing a drop in March. However, the numbers started increasing from April with a spike in July before dropping to zero from October to December (Figure 11).

Figure 11: Number of children aged 6-59 months referred for Vitamin A supplementation

HS Utilization

The number of immunization defaulters who were referred was almost the same as in 2018 from January to April but lower than the numbers in 2019. In 2020, a gradual increase was noted from July to November (Figure 12).

Figure 12: Number of immunization defaulters referred

The number of children aged zero to eleven months who were referred for immunization in 2020 was similar to those recorded in 2018 and 2019 for the months of January to February. However, in 2020, the numbers increased more than in the previous years from May to December (Figure 13).

Figure 13: Number of children aged 0-11 months referred for immunization

The number of households that reported to have hand washing facilities was higher in 2020 with the numbers increasing from may to July 2020. There was, however, a sharp drop in August although the numbers were still higher than in either 2018 or 2019 (Figure 14).

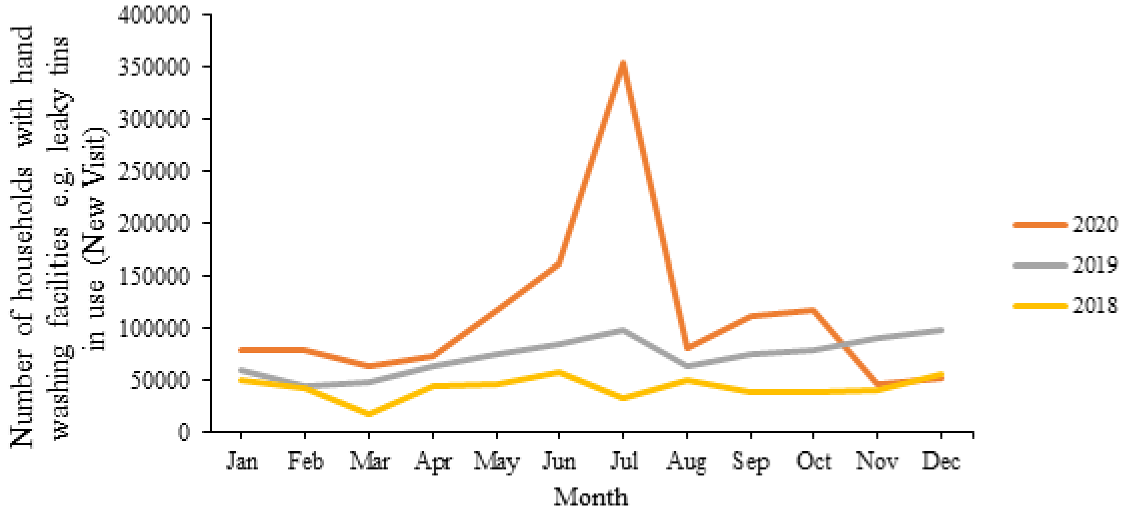

Figure 14: Number of households with hand washing facilities (New Visit)

The number of new households that were visited that had access to safe water had similar trends from January to March before the numbers increased in 2020 from April to July a trend that was not noted 2018 and 2019. The numbers later dropped from August to December 2020 (Figure 15).

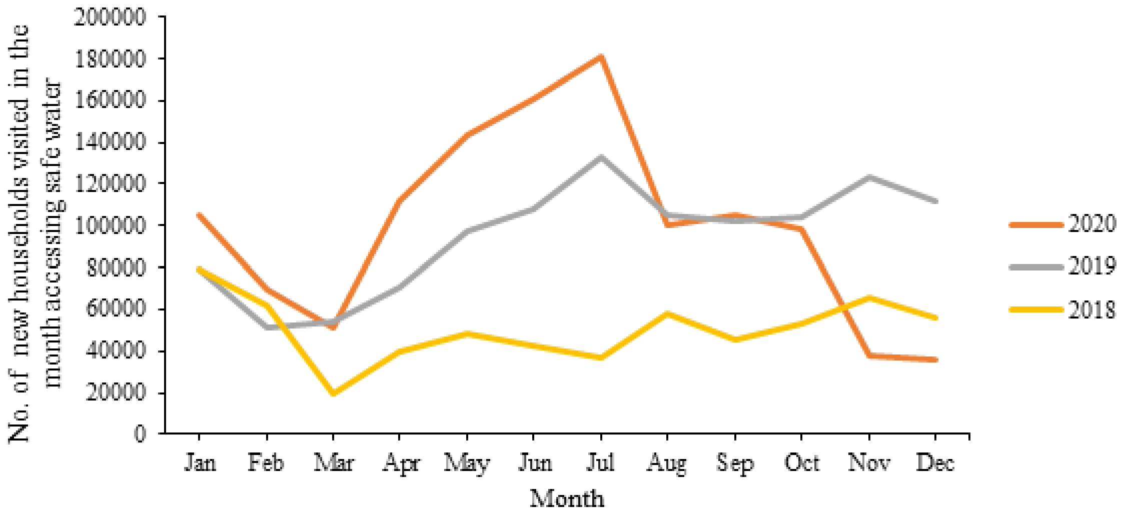

Figure 15: No. of new households visited in the month accessing safe water
