# Supplementary material for: Community health volunteers’ experiences during the COVID-19 pandemic in Kiambu county, Kenya: A qualitative study
Source: PLoS One. 2025 May 7;20(5):e0322642. doi: 10.1371/journal.pone.0322642 (PMC12057936; doi:10.1371/journal.pone.0322642)
Supplement: Attachment — Submitted filename: The Comments.docx [file pone.0322642.s003.docx]

**Community health volunteers’ experiences during the COVID-19 pandemic in Kiambu county, Kenya: a qualitative study**

**GENERAL COMMENT.**

This is generally a good research manuscript. The choice of the study population is appropriate, and the context of investigation is also very applicable. There are documented facts that COVID-19 pandemic revealed the weaknesses in the health systems across WHO African countries and most especially its implications regarding the human resources for health (HRH) is palpable. The inclusion of the CHVs in Kenya was supposed to be helpful in taming the scourge, but this research is an eye opener to the challenges associated with their duties and the gaps in governance in general at the study location. The comments below should be addressed by authors and if this is done dutifully, the manuscript can be accepted by the journal for publication.

**SECTIONAL COMMENTS**

- Line 22-23: You may want to use another suitable word here…. like ‘**CHVs participated in responding to the crisis’…….**
- Line 24-25: Expunge this phrase *‘Given that the test of a healthcare system is a health crisis*….it is not necessary
- Line 30 -31: A county in Kenya is a big geographical area with sizeable population covered by appreciable number of CHVs. Please clarify the reason behind limiting the FGDs to only 2. Ensuring saturation of information in any qualitative study is very key to avoid bias in findings
- Line 40: The word ‘disappointed’ should be replaced with another suitable word. It looks like the researcher was emotionally drawing a conclusion and may not necessarily represent the findings from the study
- Line 94: On a general note, data collection in qualitative study should harness multiple sources of information, including Focused Group Discussion (FGD), Key Informant Interview (KII) and possibly document review. This will facilitate information richness and reduce bias to the barest minimum. However, if only FGD was to be used, it was expected that it would be done with multiple groups with adoption of saturation principle. Another acceptable option is to conduct interview for reasonable sample (with applicable sampling formular) of the CHVs using self-administered semi-structured questionnaire. I’d advise that the authors consider this suggestion as important.
- Line 100: It will be good if the researcher could add other related county statistics as regards the number of health facilities, health workers/population ratio, and possibly the prevalence of the COVID-19 along with case fatality rate in the county as of the study period.
- Line 128-129: Previous pandemic or COVID-19? If for the COVID-19, for how long before the time of their selection?
- Line 151-152: You may just replace this with…..**’Two of the research-authors’**. Then how were you able to circumvent bias in the qualitative data collection knowing fully well that two of the authors served as data collectors?
- Line 154-164: The researcher should try to summarize what the FGD questions are all about. There is no need to itemize all of them
- Line 170-171: Please refer to my earlier comment on this in your abstract. Why limited to 2 FGD only, when saturation level needs to be achieved?
- Line 184: Write **PI** in full.
- Line 192: If there was no previous description of MAXQDA2020, the authors may want to add few lines to clarify that
- Line 197: Where is The University of Eastern Africa Baraton located? Is it in the same county for study location? If not, why the University?
- Line 211: Perspective of the community on what?
- Line 22-267: The author should check for full quotation of the respondent’s feedback. I guess the mother has a child with a sickness that was attended to in 7 months. In addition, the phrase ‘I removed her’ should be rephrased as appropriate
- Line 231; The quote is too short/. Author should Add a preceding statement to make the quote more comprehensive. Something must have been reported as a positive before the respondent is filled with joy.
- Line 315-316: The right way to report should be ….’The respondents reported that there was stigmatization’…..
- Line 324: Use ‘According to CHVs’ opinion….
- Line 327-328: As this statement ;323	‘*’****The political leaders themselves did not adhere to the protocols***’’ was the perception of the CHVs, the author should reflect this tone as required. A good option is to rephrase as much as possible
- Line 408; Authors can replace with ‘’’This is in tandem with the findings from studies conducted…..’
- Line 418-425: This is good. However, I expect to see other side of the arguments where other researchers’ findings contradicts this study’s findings, and they give a balanced few-lines discussion around this. This applicable to other sub-sections of the discussion
- Line 427: This phrase sound biased. One county can not stand alone to conclude that the government is disappointing. The author should find another word/phrase to convey the minds of the respondents and should not report with emotions.
- Line 428: The author may want to use another word apart from ‘pessimistic’ ...Another word like ‘respondents expressed reservations’…….
- Line 444: On a general note, the recommendation section should be summarized by the authors. Maximum of 3 paragraphs should be adequate to summarize the salient recommendations.
- Lines 445-447: Authors should not insinuate that the findings are generalisable to the whole country of Kenya. I guess a possible recommendation should be that a possible comparative study using broader method should be done across multiple counties. A better inference can be drawn if this is done in the future.
