# Supplementary material for: Community health volunteers’ experiences during the COVID-19 pandemic in Kiambu county, Kenya: A qualitative study
Source: PLoS One. 2025 May 7;20(5):e0322642. doi: 10.1371/journal.pone.0322642 (PMC12057936; doi:10.1371/journal.pone.0322642)
Supplement: Attachment — Submitted filename: Response to Reviewers 2.docx [file pone.0322642.s005.docx]

**Editor-in-Chief**

**PLOS One**

**9th January 2025**

**Dear Editor-in-Chief,**

**I hope this letter finds you well. I am writing to provide our response to the reviewers' comments on our manuscript PONE-D-24-48602 “Community health volunteers’ experiences during the COVID-19 pandemic in Kiambu county, Kenya” submitted to PLOS One.**

**In response to the reviewers' comments, we have made the relevant revisions to our work. We believe that these revisions address the concerns raised by the reviewers and strengthen the clarity and focus of our manuscript.**

**We also take this opportunity to thank the reviewers and the editor for your invaluable support and critique that has undoubtedly improved the quality of our work.**

**Thank you for considering our manuscript for publication in PLOS One. We look forward to hearing from you regarding the next steps in the review process.**

**Yours sincerely,**

**Prabhjot Kaur Juttla.**

**Editor’s comments**

**We note that this data set consists of interview transcripts. Can you please confirm that all participants gave consent for interview transcript to be published?

If they DID provide consent for these transcripts to be published, please also confirm that the transcripts do not contain any potentially identifying information (or let us know if the participants consented to having their personal details published and made publicly available). We consider the following details to be identifying information:

- Names, nicknames, and initials
- Age more specific than round numbers
- GPS coordinates, physical addresses, IP addresses, email addresses
- Information in small sample sizes (e.g. 40 students from X class in X year at X university)
- Specific dates (e.g. visit dates, interview dates)
- ID numbers

Or, if the participants DID NOT provide consent for these transcripts to be published:
- Provide a de-identified version of the data or excerpts of interview responses
- Provide information regarding how these transcripts can be accessed by researchers who meet the criteria for access to confidential data, including:
a) the grounds for restriction
b) the name of the ethics committee, Institutional Review Board, or third-party organization that is imposing sharing restrictions on the data
c) a non-author, institutional point of contact that is able to field data access queries, in the interest of maintaining long-term data accessibility.
d) Any relevant data set names, URLs, DOIs, etc. that an independent researcher would need in order to request your minimal data set.**

**Author’s response: Thank you for your valuable observation. All participants were fully informed before the study began that the data, including interview transcripts, would be published as part of the research findings. This information was clearly communicated to them during the consent process, and all participants provided explicit, signed consent.**

**We also confirm that the transcripts have been thoroughly de-identified to ensure that no individual participant can be traced or identified. Specific care was taken to remove all potentially identifying information, including names, nicknames, initials, precise ages, geographic data, physical or email addresses, dates, and any other personal identifiers. This ensures full compliance with ethical guidelines for the protection of participant confidentiality.**

**Comments to the Author**

**Reviewer #1:**

**Abstract
1. The conclusion was poorly written and should be revised.**

**Response: We have carefully revised the conclusion for improvement. Please have a look at it and thank you for this observation.**

**Main Manuscript
2. The introduction aspect of the paper needs to be reorganized to ensure flow and sequence. In addition, the authors were not convincing in their justification or rationale for this study.**

**Author’s response: Thank you for your feedback. We have completely revised the introduction to improve its structure, ensuring a more logical flow and better sequence. Additionally, we have strengthened the justification and rationale for the study.**

**Kindly find the new introduction below:**

**INTRODUCTION**

**Community health volunteers (CHVs) are recognized by the World Health Organization (WHO) as "a variety of community health auxiliaries who work in their own communities" (1). These volunteers play a critical role in advancing universal health coverage (UHC), particularly in low- and middle-income countries (LMICs) where access to essential healthcare services remains a significant challenge (2,3). In these settings, UHC relies heavily on the extension of primary health care (PHC) into local communities, with CHVs serving as the first point of contact for healthcare delivery and contributing to the effectiveness of healthcare systems (4).**

**In Kenya, CHVs are key participants in the national community health strategy, which is designed to promote community involvement in healthcare decision-making and delivery. The strategy is managed by community health strategy coordinators (CHSCs), who oversee CHV activities and ensure that volunteers have access to the necessary tools and resources for service provision. CHVs are entrusted with the important task of promoting preventive health measures, supporting health education, and ensuring the continuity of care, especially at the grassroots level (10).**

**Historically, CHVs in Kenya operated in an informal capacity, without formal recognition or integration into the national healthcare system (11). Before the enactment of the Public Health Act in October 2023, CHVs functioned as volunteers, largely driven by local initiatives and their personal commitment to improving public health (12). Their roles, which primarily involved delivering health information and encouraging healthcare-seeking behaviour, were ad hoc and lacked structure (11). Training for CHVs, when provided, was inconsistent and varied across regions, without standardized guidelines to ensure uniformity in knowledge and skill. Moreover, these volunteers often operated without compensation, relying on their dedication to improving health outcomes in underserved areas.**

**The outbreak of the COVID-19 pandemic further highlighted the importance of CHVs in the Kenyan healthcare system. Since the first confirmed case in March 2020 (5), the pandemic disrupted healthcare systems globally, and Kenya was no exception. In response, CHVs were enlisted as vital participants in Kenya’s COVID-19 Response Strategy (5,8). Their roles expanded to include promoting behavioral changes, combating misinformation, encouraging hygienic practices, and sustaining efforts to address other priority diseases at the community level (7,8). CHVs also contributed to contact tracing efforts and monitored vulnerabilities within their communities. Despite these critical contributions, CHVs continued to work as volunteers throughout the pandemic, depending on the government, through CHSCs, for the provision of personal protective equipment (PPE), sanitizers, and other necessary supplies (10). They were also expected to receive psychosocial support to help manage the increased demands of their roles. Unfortunately, these measures were often insufficient, underscoring gaps in Kenya’s healthcare system and raising concerns about its preparedness and resilience during health crises.**

**Given the ongoing legislative reforms aimed at strengthening Kenya’s healthcare system and advancing UHC (9), it is crucial to examine the experiences of CHVs during the pandemic. This study seeks to explore the challenges faced by CHVs and the community members they served while implementing COVID-19 control measures. By analyzing their lived experiences and evaluating their perspectives on government crisis management at the community level, this research offers insights into the role of CHVs and contributes to the broader discourse on improving healthcare system preparedness in LMICs.**

**3. Which kind of support was provided to the CHVs prior to the promulgation of law that recognized their role in the health system?**

**Response: Thank you for this observation. We have detailed the same in the introduction section of the manuscript above, and this is a key detail regarding the CHVs past roles and responsibilities and reflects their condition at the time of the study.**

**4. How many health facilities are this county, and what kind of services do they provide, especially during the COVID-19 pandemic?**

**Response: we have added this information to the study setting section of our study**

**“****At the time of the study, the county was served by a total of 98 health facilities, distributed across various levels of care as follows: three Tier 5 inter-county facilities, 13 Tier 4 hospitals, 24 Tier 3 health centres, and 70 Tier 2 dispensaries. The number of Level 1 facilities is consistent with the above distribution. During the COVID-19 pandemic, the county adopted a centralized approach to managing COVID-19 cases by designating Tigoni Level 4 Hospital as the exclusive facility for COVID-19 services. Consequently, all COVID-19 patients were referred to this hospital at the time of the study.”**

**5. What is/are the roles of the CHVs (not CHPs) played in relation to these HFs or various levels of care, if any?**

**Author’s response: Before 2023, Community Health Volunteers (CHVs) in Kenya played a critical (but rudimentary) role in bridging the gap between communities and healthcare facilities across various levels of care, despite their informal status and limited recognition within the formal health system. They were essential in promoting health and raising basic awareness by educating community members on disease prevention, hygiene, nutrition, and maternal and child health. CHVs encouraged the utilization of health facilities for preventive and curative services, fostering trust and knowledge about available healthcare resources. This has been mentioned in our introduction section.**

**6. I suggest the study population and sampling should come after the study design.**

**Author’s response: Thank you for this observation. I have placed the study population and sampling after the study design.**

**7. What do you mean by this: “The data was collected as part of a larger quasi-experimental mixed methods pre-post study”?**

**Author’s response: This paper on Community Health Volunteers (CHVs) was part of a broader study examining the impact of COVID-19 on Level 1 healthcare services. The study employed multiple methodologies, including focus group discussions (FGDs), a desk review conducted by the Health Records and Information Officer (HRIO) in Kiambu County, and key informant interviews with members of the County Health Management Team (CHMT). The desk review revealed critical challenges faced by CHV units, including their limited numbers, high attrition rates, and inadequate access to essential commodities.**

**During the FGDs, findings from the desk review were presented to the CHVs for validation. However, the CHVs strongly disowned the data, expressing confusion and stating that they did not recognize or understand the data reported in the Kenya Health Information System (KHIS). This raised concerns about the reliability of the desk review findings and the potential disconnect between reported data and ground-level realities. We have included this desk review as Supplementary Material File S2. Additionally, while key informant interviews were conducted with CHMT leaders, their responses were inconsistent and varied widely, making it difficult to derive actionable insights from this component of the study. However, we have now expunged this phrase.**

**8. The selection of CHVs was somewhat opaque and unspecific. Can you please elaborate on the selection of CHVs for the FGD sessions?**

**Author’s Response: Thank you for raising this important point. To determine the number of CHVs in Kiambu County eligible for selection, the study examined the functional Community Health Units (CHUs) within the county from 2017 to 2020, as shown in the table 1 below, derived from the Kenya Health Information System (KHIS).**

**SAMPLING**

**Therefore, to determine the number of CHVs in Kiambu County eligible for selection, the study examined the functional Community Health Units (CHUs) within the county from 2017 to 2020, as shown in Table 1 below.**

***Table 1: The total number of functional CHUs within Kiambu County during the period under study. The data was derived from the Kenya Health Information System (KHIS).***

| Year | Jan | Feb | Mar | Apr | May | Jun | Jul | Aug | Sept | Oct | Nov | Dec |
| --- | --- | --- | --- | --- | --- | --- | --- | --- | --- | --- | --- | --- |
| 2017 | 8 | 3 | 3 | 7 | 7 | 3 | 3 | 8 | 1 | 2 | 0 | 0 |
| 2018 | 0 | 3 | 0 | 0 | 6 | 0 | 0 | 0 | 0 | 0 | 0 | 3 |
| 2019 | 1 | 7 | 12 | 19 | 17 | 26 | 8 | 6 | 11 | 11 | 12 | 19 |
| 2020 | 19 | 15 | 1 | 6 | 6 | 6 | 10 | 31 | 77 | 0 | 0 | 0 |

**To participate in the FGDs, CHVs were required to meet the inclusion criterion below.**

**Inclusion criteria: To be eligible for participation in the FGD, a CHV had to have at least 5 years of experience, including the three years within the study period (2017–2020), have been actively involved in the pandemic response, and be available for the interview.**

**Exclusion criteria: Any CHV who did not meet the above stipulated qualifications was excluded.**

**However, identifying eligible CHVs was challenging due to the consistently low number of functional CHUs prior to 2020 (Table 1). The substantial increase in functional CHUs and the heightened engagement of CHVs observed in 2020 reflects the increased demand for community health services driven by the COVID-19 pandemic and the GOK community health strategy during this period. After consultations with CHSCs, it was determined that only 50 CHVs met the inclusion criteria.**

**Participants were selected through convenience sampling. The pool of eligible participants was restricted to 50 CHVs who met the inclusion criteria, ensuring a basis for comparison of experiences before and during the pandemic. Two FGDs were conducted: one comprising 12 women and the other comprising 12 men, resulting in a sample size that represented 48% of the eligible CHVs.**

**To ensure adequate representation of Kiambu County, which consists of 12 sub-counties: two CHVs were selected from each sub-county. This included 12 participants from upper Kiambu, a predominantly rural area, and 12 participants from lower Kiambu, which is more urbanized.**

**9. How was the desired number of participants determined?**

**Author’s response: Kindly note our response above.**

**10. Line 139, why do you have to use the word exigencies? I suggest you use the word needs or requirements**

**Author’s response: we have used the word “Needs” instead. Thank you for this.**

**11. Were the participants informed of their preserved right to quit the study at any time? Was it verbal or in writing?**

**Author’s response: We have clarified the same as below “The participants were free to leave at any time during the FGD and this was communicated in the consent form.”**

**12. The PI (MMK) was the County Head of Clinical Research while the researcher (NW) who did data collection NW was the Monitoring and Evaluation Coordinator. Can you please re-write this?**

**Author’s response: We have rewritten this as below for additional clarity: “The Principal Investigator (PI), MMK, served as the County Head of Clinical Research. The researcher, NW, who conducted the data collection, was the Monitoring and Evaluation Coordinator. The transcriber, PJ, has extensive experience in transcribing qualitative data.”**

**13. There seem to be contradictions in the emerging ideas from the FGDs considering the barriers and their roles.**

**Author’s response: Thank you for this observation. We have now reworked this section to remove any contradicting ideas and stuck to the physical barriers to performing their duties, as below:**

### Barriers to community health service delivery

**The COVID-19 pandemic disrupted the ability of CHVs to perform their duties due to the implementation of public health measures aimed at curbing the spread of the virus One of the key strategies employed by the government was the restriction of physical movement through the establishment of zonal roadblocks on major public routes. These restrictions had far-reaching consequences, not only limiting patients' access to healthcare facilities for regular appointments but also restricting CHVs’ ability to reach their clients. This was particularly problematic for individuals with chronic conditions, such as those attending comprehensive care clinics (CCC) for HIV treatment.**

***“As a community volunteer … we didn’t go door-to-door to check for the clients that we had. We didn’t have equipment that we should have used for that period. And still, the community that needed us didn’t have any gathering [due to social distancing] to educate them in various ways.” (Resp.11)***

***“…like those patients who would come to CCC, many were far away and there were roadblocks. For instance, maybe they’re from Lari and they come to the facility in Tigoni, and now there was a road block at Mathore. So now will they really pass through?” (Resp.12)***

***“okay to add to that I can say that most of the community members found that since there was cessation of movement most transport or public means were not available to them. So, it was required to take a taxi or a motorbike and then also the fare was hiked. Then you get to the roadblock and you asked where you are going and you remove a paper from the hospital and say I am going to hospital. So, to avoid all that you just stay at home.” (Resp.7)***

**In addition to the movement restrictions and roadblocks, a curfew was imposed, further restricting people's ability to seek emergency care outside of designated hours. As a result, even though alternative channels for emergency medical services were put in place, these measures were not widely known or accessible. This compounded the challenges of timely response to emergencies, as individuals were unable to receive prompt care during the restricted hours.**

***“Because curfew was enforced, even motorbikes didn’t go anywhere, cars were not on the road. To have an illness meant you had a lot of problems and not all of the people knew that 1196 number. Many people died because they didn’t get access to doctors. they just died.” (Resp.3)***

**When CHVs were able to visit patients at their homes, they faced additional challenges in providing care due to the need to observe social distancing and adhere to the government’s ‘low touch’ guidelines. This was further compounded by the lack of appropriate personal protective equipment (PPE) and essential Point of Care Diagnostic (PoCD) devices.**

***“You had to somehow keep distance while measuring that child’s temperature, and then you had to sanitize everything you used. Then the parents also had to sanitize. it was even better if you showed the mother how to check the temperature and they did it for you. Then after the mother checked the temperature you had to sanitize it again. It was hard work for parents to understand what you are saying, you see you are not close to them…” (Resp.8)***

**14. At what point in the COVID-19 pandemic were the CHVs provided with kits, such as face masks, by the NGO- Living Goods, given that a major limitation to service delivery by the CHVs was the lack of appropriate PPE and basic devices at the point of care?**

**Author’s response: Masks posed a significant challenge, particularly during the initial stages of the COVID-19 pandemic when access to personal protective equipment (PPE) was severely limited. However, Living Goods promptly addressed this issue by providing masks to CHVs within the second or third month following the onset of the pandemic.**

**15. This is in keeping with studies conducted in Guinea, Liberia and Sierra Leone during the Ebola outbreak (23). How many studies have you cited here? Kindly correct this.**

**Author’s response: Thank you for this insightful observation. It is important to note that this study was a single comprehensive effort that collected and integrated data from all three countries into one cohesive paper, as detailed in the following citation:**

**Miller NP, Milsom P, Johnson G, Bedford J, Kapeu AS, Diallo AO, et al. Community health workers during the Ebola outbreak in Guinea, Liberia, and Sierra Leone. J Glob Health. 2018;8(2).**

**16. “The present study also highlighted the negative economic effects of the response to COVID-19 on the community members. This is corroborated by another study conducted in Poland where people relayed similar financial destitution as a result of the pandemic measures (25).” This does not tally with the sub-topic of misinformation or being scared about the disease. Perhaps, this (economic implication) can be discussed elsewhere.**

**Author’s response: Thank you for this observation. We have noted the same and moved the discussion for the latter under a new sub-heading “Economic hardship”.**

**17. Regarding disappointment in government, the authors should discuss what they found (results) in the current study, then compare it with other studies (local and international) and proffer possible explanations, where relevant.**

**Author’s response: Thank you for the suggestion. We have revised the manuscript to include a more detailed discussion of the findings from our study and have expanded the comparison with both local and international studies. Additionally, we have provided relevant explanations for the observed results based on contextual factors and existing literature.**

**18. The discussion requires a lot of improvement as it seems shallow and unconvincing.**

**Author’s response: Thank you for your valuable feedback. We have made substantial improvements to the discussion section, providing a deeper analysis and clearer connections between our findings and existing research. We hope the revised discussion now presents a more comprehensive and convincing argument.**

**19. There are other real and potential limitations and biases in the study that should have been stated in the manuscript. The authors need to think through this.**

**Author’s response: Thank you for pointing this out. We have carefully reviewed and considered additional limitations and biases in the study. These have now been explicitly stated in the manuscript, and we have provided further strengths to consider in view of these limitations.**

**20. The entire manuscript requires editing.**

**Author’s response: Thank you for your feedback. We have thoroughly revised the entire manuscript for clarity, coherence, and conciseness. We have made substantial edits to improve the flow of ideas and ensure that the manuscript meets the highest academic standards.**

**21. On the recommendation, this study was conducted before the CHVs (community health strategy) were formally integrated into and compensated by the formal health system by the GoK. Hence, how does this study adequately identify the challenges and obstacles towards realizing the UHC goal in the context of the roles of the CHVs given the temporal change(s)?**

**Author’s response: Thank you for pointing this out. We acknowledge the temporal changes that have occurred since the study was conducted, specifically the formal integration and compensation of CHVs by the Government of Kenya (GoK). The study highlights the challenges and obstacles faced by CHVs during the study period, which were primarily rooted in the lack of recognition and resources. While acknowledging that the landscape has changed since the study, we believe the findings remain valuable as they provide a snapshot of the CHVs' challenges before formal compensation, which can still inform current discussions and improvements in the community health strategy.**

**22. The recommendations are lengthy and somewhat boring. The authors should summarise and make this section concise.**

**Author’s response: We appreciate your feedback regarding the length and presentation of the recommendations section. As per your suggestion, we have summarized and condensed the recommendations to make the section more concise and engaging. We aimed to highlight the most critical points that align with the study's findings while maintaining clarity and brevity in the text.**

**23. The discussion, conclusion and recommendation require considerable revision with a clear flow of thought and reorganization, in alignment with the objectives of the study.**

**Author’s response: Thank you for your comments on the discussion, conclusion, and recommendations sections. We have revised these sections for better coherence, ensuring they are clearly aligned with the objectives of the study. The flow of thought has been reorganized to make the connections between findings, implications, and recommendations more explicit and logically structured. This revision aims to provide a clearer and more impactful presentation of the study's key outcomes.**

# **Reviewer #2:**

Community health volunteers’ experiences during the COVID-19 pandemic in Kiambu county, Kenya: a qualitative study

GENERAL COMMENT.
This is generally a good research manuscript. The choice of the study population is appropriate, and the context of investigation is also very applicable. There are documented facts that COVID-19 pandemic revealed the weaknesses in the health systems across WHO African countries and most especially its implications regarding the human resources for health (HRH) is palpable. The inclusion of the CHVs in Kenya was supposed to be helpful in taming the scourge, but this research is an eye opener to the challenges associated with their duties and the gaps in governance in general at the study location. The comments below should be addressed by authors and if this is done dutifully, the manuscript can be accepted by the journal for publication.

**Author’s response: Thank you very much for your thoughtful and encouraging feedback. We truly appreciate your recognition of the relevance and applicability of our study, as well as the insight you provided regarding the broader context of the COVID-19 pandemic's impact on health systems, particularly in Africa.**

**SECTIONAL COMMENTS
Line 22-23: You may want to use another suitable word here…. like ‘CHVs participated in responding to the crisis’…….**

**Author’s response: We have revised the phrase to: “To cushion against this potential effect in Kenya, community health volunteers (CHVs) participated by responding to the crisis by delivering their usual community-based health services and added specific roles in regards to COVID-19 mitigation.”**

**Line 24-25: Expunge this phrase ‘Given that the test of a healthcare system is a health crisis….it is not necessary**

**Author’s response: The phrase on lines 24-25 has been removed as requested.**

**Line 30 -31: A county in Kenya is a big geographical area with sizeable population covered by appreciable number of CHVs. Please clarify the reason behind limiting the FGDs to only 2. Ensuring saturation of information in any qualitative study is very key to avoid bias in findings**

**Author’s response: Thank you for this observation. We have expounded on this reasoning in our “Sampling” section of our manuscript, as below.**

**SAMPLING**

**Therefore, to determine the number of CHVs in Kiambu County eligible for selection, the study examined the functional Community Health Units (CHUs) within the county from 2017 to 2020, as shown in Table 1 below.**

***Table 1: The total number of functional CHUs within Kiambu County during the period under study. The data was derived from the Kenya Health Information System (KHIS).***

| Year | Jan | Feb | Mar | Apr | May | Jun | Jul | Aug | Sept | Oct | Nov | Dec |
| --- | --- | --- | --- | --- | --- | --- | --- | --- | --- | --- | --- | --- |
| 2017 | 8 | 3 | 3 | 7 | 7 | 3 | 3 | 8 | 1 | 2 | 0 | 0 |
| 2018 | 0 | 3 | 0 | 0 | 6 | 0 | 0 | 0 | 0 | 0 | 0 | 3 |
| 2019 | 1 | 7 | 12 | 19 | 17 | 26 | 8 | 6 | 11 | 11 | 12 | 19 |
| 2020 | 19 | 15 | 1 | 6 | 6 | 6 | 10 | 31 | 77 | 0 | 0 | 0 |

**To participate in the FGDs, CHVs were required to meet the inclusion criterion below.**

**Inclusion criteria: To be eligible for participation in the FGD, a CHV had to have at least 5 years of experience, including the three years within the study period (2017–2020), have been actively involved in the pandemic response, and be available for the interview.**

**Exclusion criteria: Any CHV who did not meet the above stipulated qualifications was excluded.**

**However, identifying eligible CHVs was challenging due to the consistently low number of functional CHUs prior to 2020 (Table 1). The substantial increase in functional CHUs and the heightened engagement of CHVs observed in 2020 reflects the increased demand for community health services driven by the COVID-19 pandemic and the GOK community health strategy during this period. After consultations with CHSCs, it was determined that only 50 CHVs met the inclusion criteria.**

**Participants were selected through convenience sampling. The pool of eligible participants was restricted to 50 CHVs who met the inclusion criteria, ensuring a basis for comparison of experiences before and during the pandemic. Two FGDs were conducted: one comprising 12 women and the other comprising 12 men, resulting in a sample size that represented 48% of the eligible CHVs.**

**To ensure adequate representation of Kiambu County, which consists of 12 sub-counties: two CHVs were selected from each sub-county. This included 12 participants from upper Kiambu, a predominantly rural area, and 12 participants from lower Kiambu, which is more urbanized.**

**Line 40: The word ‘disappointed’ should be replaced with another suitable word. It looks like the researcher was emotionally drawing a conclusion and may not necessarily represent the findings from the study**

**Author’s Response: Removed the word “disappointed” as below –**

**The CHVs were skeptical in the county’s preparedness in dealing with both the COVID-19 and future pandemics.**

**Line 94: On a general note, data collection in qualitative study should harness multiple sources of information, including Focused Group Discussion (FGD), Key Informant Interview (KII) and possibly document review. This will facilitate information richness and reduce bias to the barest minimum. However, if only FGD was to be used, it was expected that it would be done with multiple groups with adoption of saturation principle. Another acceptable option is to conduct interview for reasonable sample (with applicable sampling formular) of the CHVs using self-administered semi-structured questionnaire. I’d advise that the authors consider this suggestion as important.**

**Author’s Response: This study on Community Health Volunteers (CHVs) was part of a larger investigation into the impact of COVID-19 on Level 1 healthcare services. The research employed multiple methods, including focus group discussions (FGDs), a desk review conducted by the Health Records and Information Officer (HRIO) which was extracted from the KHIS (Supplementary Material 2), and key informant interviews with members of the County Health Management Team (CHMT).**

**The desk review revealed significant challenges faced by CHV units, such as their limited numbers, high attrition rates, and inadequate access to essential commodities. However, when the desk review findings were presented to the CHVs during the FGDs, they disowned the data, stating that they did not understand the information reported in the KHIS. This raised concerns about the reliability of the desk review data and highlighted a potential disconnect between the reported statistics and ground-level realities. Additionally, while key informant interviews were conducted with CHMT leaders, their responses were inconsistent and varied widely, making it difficult to derive actionable insights from this component of the study. As a result, the study encountered significant challenges in reconciling the data sources and interpreting the findings.**

**SAMPLING**

**Therefore, to determine the number of CHVs in Kiambu County eligible for selection, the study examined the functional Community Health Units (CHUs) within the county from 2017 to 2020, as shown in Table 1 below.**

***Table 1: The total number of functional CHUs within Kiambu County during the period under study. The data was derived from the Kenya Health Information System (KHIS).***

| Year | Jan | Feb | Mar | Apr | May | Jun | Jul | Aug | Sept | Oct | Nov | Dec |
| --- | --- | --- | --- | --- | --- | --- | --- | --- | --- | --- | --- | --- |
| 2017 | 8 | 3 | 3 | 7 | 7 | 3 | 3 | 8 | 1 | 2 | 0 | 0 |
| 2018 | 0 | 3 | 0 | 0 | 6 | 0 | 0 | 0 | 0 | 0 | 0 | 3 |
| 2019 | 1 | 7 | 12 | 19 | 17 | 26 | 8 | 6 | 11 | 11 | 12 | 19 |
| 2020 | 19 | 15 | 1 | 6 | 6 | 6 | 10 | 31 | 77 | 0 | 0 | 0 |

**To participate in the FGDs, CHVs were required to meet the inclusion criterion below.**

**Inclusion criteria: To be eligible for participation in the FGD, a CHV had to have at least 5 years of experience, including the three years within the study period (2017–2020), have been actively involved in the pandemic response, and be available for the interview.**

**Exclusion criteria: Any CHV who did not meet the above stipulated qualifications was excluded.**

**However, identifying eligible CHVs was challenging due to the consistently low number of functional CHUs prior to 2020 (Table 1). The substantial increase in functional CHUs and the heightened engagement of CHVs observed in 2020 reflects the increased demand for community health services driven by the COVID-19 pandemic and the GOK community health strategy during this period. After consultations with CHSCs, it was determined that only 50 CHVs met the inclusion criteria.**

**Participants were selected through convenience sampling. The pool of eligible participants was restricted to 50 CHVs who met the inclusion criteria, ensuring a basis for comparison of experiences before and during the pandemic. Two FGDs were conducted: one comprising 12 women and the other comprising 12 men, resulting in a sample size that represented 48% of the eligible CHVs.**

**To ensure adequate representation of Kiambu County, which consists of 12 sub-counties: two CHVs were selected from each sub-county. This included 12 participants from upper Kiambu, a predominantly rural area, and 12 participants from lower Kiambu, which is more urbanized.**

**Line 100: It will be good if the researcher could add other related county statistics as regards the number of health facilities, health workers/population ratio, and possibly the prevalence of the COVID-19 along with case fatality rate in the county as of the study period.**

**Author’s response: Thank you for this. we have included this information in the study settings section of our manuscript, as below.**

**Study setting**

**The study was conducted in Kiambu County, located in the central region of Kenya, covering an area of 2,543.5 km². According to the 2019 census, the average household size in the county is five individuals. At the time of the study, Kiambu had 795,241 households, 132 community units, and an estimated 3,085 Community Health Volunteers (CHVs). The CHV-to-household ratio was 1:100, and the community unit-to-household ratio was 1:1,000 (5). The county was served by 98 health facilities distributed across various levels of care, including three Tier 5 inter-county facilities, 13 Tier 4 hospitals, 24 Tier 3 health centres, and 70 Tier 2 dispensaries.**

**During the COVID-19 pandemic, Kiambu adopted a centralized approach to managing COVID-19 cases, designating Tigoni Level 4 Hospital as the sole facility for COVID-19 services (12). As a result, all COVID-19 patients were referred to this hospital during the study period. At the time of the study, Kiambu County had the second-highest number of confirmed COVID-19 cases in Kenya (13). The county reported its first COVID-19 case in March 2020, and by August 2022, the total number of confirmed cases had risen to 21,209, with 896 deaths (13), resulting in a case-fatality rate of 4.22%.**

**Line 128-129: Previous pandemic or COVID-19? If for the COVID-19, for how long before the time of their selection?**

**Author’s response: we have edited our inclusion criteria for clarity, as below:-**

**“*Inclusion criteria:* To be eligible for participation, a CHV had to have at least 5 years of experience, including the three years within the study period (2017–2020), have been actively involved in the pandemic response, and be available for the interview.”**

**Line 151-152: You may just replace this with…..’Two of the research-authors’. Then how were you able to circumvent bias in the qualitative data collection knowing fully well that two of the authors served as data collectors?**

**Author’s response: For the sake of transparency, we believe it is important to clearly specify the roles of each member of the research team during the study. Two of the authors were trained and served as data collectors, but only one of them participated in the analysis of the data. The training was designed to ensure objectivity and minimize any potential bias during the data collection process. Additionally, rigorous protocols were followed throughout the study to maintain the integrity of the data and ensure that the findings were based solely on the input of the participants. This approach is consistent with standard research practices to mitigate any influence on the data collection process.**

**Line 154-164: The researcher should try to summarize what the FGD questions are all about. There is no need to itemize all of them**

**Author’s response: In the interest of transparency and to support replication studies, we have included the specific questions asked during the FGDs. However, we understand the importance of summarizing the main themes or focus areas of the questions, and hence we have summarized the questions while maintaining their core meaning in the manuscript text.**

**Line 170-171: Please refer to my earlier comment on this in your abstract. Why limited to 2 FGD only, when saturation level needs to be achieved?**

**Author’s response: Kindly see our response to this comment, as above.**

**Line 184: Write PI in full.**

**Author’s response: we have written PI (principal investigator) in full in the text. Thank you.**

**Line 192: If there was no previous description of MAXQDA2020, the authors may want to add few lines to clarify that**

**Author’s response: Thank you for this. we have clarified the use of the software in the manuscript text as below:**

**“For added rigor, the agreed-upon codes were used to classify segments of the transcripts using MAXQDA 2020, a qualitative data analysis software that facilitates systematic coding, categorization, and organization of textual data. The software provides tools to quantify how often a particular code was mentioned, helping to identify patterns in the data corpus. Any discrepancies in the coding process were resolved through discussions among MMK, NW, and PJ, who collaboratively agreed on the emergent themes.”**

**Line 197: Where is The University of Eastern Africa Baraton located? Is it in the same county for study location? If not, why the University?**

**Author’s response: The University of Eastern Africa, Baraton (UEAB) is located in Kenya. In addition to obtaining county approvals and the research license for the study, we sought a scientific review and ethical approval, which could be facilitated by the university. This process ensured the study adhered to ethical standards and received the necessary academic oversight.**

**Line 211: Perspective of the community on what?**

**Author’s response: Thank you for the clarification. One of the questions we asked focused on the community's perspective of COVID-19, including how it is spread and the prevention measures. This was aimed at obtaining surrogate responses from community members through the CHVs on their views regarding these issues.**

**We have thus edited the phrase to reflect the same: “**Part B presents the perspectives of the community regarding COVID-19 as reported by the CHVs.”

**Line 22-267: The author should check for full quotation of the respondent’s feedback. I guess the mother has a child with a sickness that was attended to in 7 months. In addition, the phrase ‘I removed her’ should be rephrased as appropriate**

**Author’s response: Thank you for your observation. For this quote, we interpreted the context to be about ANC visits and have modified the direct quote from the CHV. The ellipsis in this context represents a pause in the respondent’s speech. The original quote “I removed her” was changed to “I convinced her to leave her house,” and this modification is reflected in square brackets as follows:**

***“People of ANC, the mothers… because I remember there was a place I went for a home visit, the mother told me she hasn’t been to the clinic in seven months. Seven months! And she hasn’t gone to the clinic. I told her ‘Let’s go, because these months you don’t know the state of your child’. [I convinced her to leave] her house and we went. I only left her once she was seen at the facility, I told her ‘Let’s not be afraid, let’s go. See even me I will be there, let’s go’. Then those women who gave birth, they were maybe in the third month and during the 4th month COVID was announced, so many skipped immunizations. They skipped period because when they were told ‘stay-at-home. Lockdown.’ They didn’t have an otherwise.” (Resp.7)***

**Line 231; The quote is too short/. Author should Add a preceding statement to make the quote more comprehensive. Something must have been reported as a positive before the respondent is filled with joy.**

**Author’s response: Thank you for this observation. We have given additional context for this quote as below:**

***“I serve* *I was able to overcome my challenges because first of all I was filled with love for the people I serve. When I would visit them, I would ensure that they would wash their hands many times, wear their masks and also that they had sanitizers.” (Resp.2)***

**Line 315-316: The right way to report should be ….’The respondents reported that there was stigmatization’…..**

**Author’s response: we have edited the phrase to read as below:**

**“The respondents reported that healthcare workers in the facilities, CHVs, sick community members, and foreigners faced stigmatization.”**

**Line 324: Use ‘According to CHVs’ opinion….**

**Author’s response: Thank you for this observation. We have edited the phrase as such “According to the CHVs’ opinions, initial adherence to COVID-19 requirements gradually decreased as a result of financial consequences.”**

**Line 327-328: As this statement ;323 ‘’The political leaders themselves did not adhere to the protocols’’ was the perception of the CHVs, the author should reflect this tone as required. A good option is to rephrase as much as possible**

**Author’s response: we greatly appreciate this guidance and have revised the paragraph as below:-**

**The general perception among the CHVs was that the government had neglected its sick citizens and failed to provide financial support during the pandemic. Additionally, gender-based violence was seen as a consequence of these financial challenges. There was also a belief that political leaders themselves did not adhere to the health protocols.**

**Line 408; Authors can replace with ‘’’This is in tandem with the findings from studies conducted…..’**

**Author’s response: we have revised the phrase as directed below**

**This is in tandem with the findings from studies conducted in Guinea, Liberia and Sierra Leone during the Ebola outbreak (24).**

**Line 418-425: This is good. However, I expect to see other side of the arguments where other researchers’ findings contradicts this study’s findings, and they give a balanced few-lines discussion around this. This applicable to other sub-sections of the discussion**

**Author’s response: Thank you for your valuable feedback. We have revised the discussion section to incorporate contrasting viewpoints and findings from other researchers. In this revision, we provide a more balanced discussion by acknowledging studies that may contradict our findings. We believe this inclusion strengthens the depth of our analysis and offers a more comprehensive view of the topic.**

**Line 427: This phrase sound biased. One county can not stand alone to conclude that the government is disappointing. The author should find another word/phrase to convey the minds of the respondents and should not report with emotions.**

**Author’s response: in order to address this bias in the subtitle, we have revised the phrase as follows: “Perceived shortcomings of the government”**

**Line 428: The author may want to use another word apart from ‘pessimistic’ ...Another word like ‘respondents expressed reservations’……**.

**Author’s response: The phrase has been changed to “****The respondents expressed reservations about the county government’s handling of COVID-19 and any potential future pandemics.”**

**Line 444: On a general note, the recommendation section should be summarized by the authors. Maximum of 3 paragraphs should be adequate to summarize the salient recommendations.**

**Author’s response: Thank you for this suggestion. We have revised the recommendations section to condense it into three concise paragraphs that summarize the key points effectively.**

**Lines 445-447: Authors should not insinuate that the findings are generalisable to the whole country of Kenya. I guess a possible recommendation should be that a possible comparative study using broader method should be done across multiple counties. A better inference can be drawn if this is done in the future.**

**Author’s response: Thank you for your feedback. We have revised the text to remove any reference to the generalizability of the findings to the entire country. We have also included this as a recommendation for future studies.**
