# Supplementary material for: Community health volunteers’ experiences during the COVID-19 pandemic in Kiambu county, Kenya: A qualitative study
Source: PLoS One. 2025 May 7;20(5):e0322642. doi: 10.1371/journal.pone.0322642 (PMC12057936; doi:10.1371/journal.pone.0322642)
Supplement: Attachment — Submitted filename: PLOS Response to Reviewers 17_03_2025.docx [file pone.0322642.s006.docx]

**Editor-in-Chief**

**PLOS One**

**17th March 2025**

**Dear Editor-in-Chief,**

**We appreciate the opportunity to revise our manuscript, “Community Health Volunteers’ Experiences During the COVID-19 Pandemic in Kiambu County, Kenya” (PONE-D-24-48602), submitted to *PLOS ONE*.**

**In response to the editor’s comments, we have carefully revised the manuscript to address the concerns raised and believe these revisions have strengthened its clarity and focus.**

**We sincerely thank the reviewers and the editor for their constructive feedback, which has undoubtedly improved the quality of our work.**

**Thank you for your time and consideration. We look forward to your feedback on the revised submission and the next steps in the review process.**

**Yours sincerely,**

**Prabhjot Kaur Juttla.**

**Editor’s comments**

**The issue of the conduction of only 2 FGDs vis-a-vis saturation of information is a challenge that you still have to address. I would suggest that you include this in the study limitation section and discuss its impact on the reliability of your findings, conclusions and recommendations.**

**Author’s response:**

Thank you for the opportunity to address this important concern. We acknowledge the limitations associated with conducting only two FGDs and have made every effort to transparently discuss its impact throughout the manuscript. Specifically, we have explicitly addressed this issue in the **abstract, study limitations, recommendations, and conclusion** sections. Furthermore, in the **sampling section**, we have provided the raw numbers of the functional units available at the time of the study, which directly constrained our sample size. Recognizing that this limitation affects the reliability and generalizability of our findings, we have carefully framed our conclusions and recommendations accordingly.

Please find the revisions highlighted in bold blue within the relevant sections following this response (below). We hope these clarifications are sufficient, and we appreciate your consideration for publication.

**Edited points in the manuscript:**

**Abstract line 32-27, and line 50-53**

**Methods:** This study employed a phenomenological exploratory qualitative design. Due to logistical constraints, only two focus group discussions could be conducted with 24 CHVs, representing 48% of eligible participants who met the inclusion criteria: a minimum of five years of experience, active involvement in the pandemic response, and availability for the interview. Data were digitally recorded, transcribed, translated, and coded for thematic analysis.

**Conclusion:** ... However, the study's limitations, particularly the small number of FGDs and lack of data saturation, should be considered when interpreting the findings. Nonetheless, it provides a valuable starting point for further research and comparative analysis across other counties in Kenya.

## **Limitations, line 540-558**

A key limitation of this study is that only two FGDs were conducted; therefore, the findings and conclusions should be interpreted with caution, given the limited extent to which data saturation was achieved. The absence of established saturation affects the reliability of the findings and constrains their broader applicability. The limited sample size was primarily due to the availability of only 48% of eligible CHVs during the COVID-19 crisis, as pandemic-related restrictions made recruitment particularly challenging. Although participants were drawn from diverse settings within Kiambu County, encompassing both lower Kiambu (urban and peri-urban areas) and upper Kiambu (rural areas), the small number of FGDs limits the extent to which these findings can be extrapolated to the broader healthcare landscape. Future research should continue data collection until a valid point of saturation is reached to enhance the depth and robustness of the findings.

Nonetheless, this study provides a preliminary foundation for future research, particularly given the longstanding neglect of CHVs and the systemic challenges they face. Across counties, Level 1 services have been largely overlooked, CHVs have experienced high attrition rates and demoralization, stipends have not been consistently provided, and the policy framework for community health services remains in the process of institutionalization (35–37). While we acknowledge the study's limitations, the insights presented here contribute to a deeper understanding of the realities faced by CHVs and underscore the need for further research and policy attention.

**Recommendations, line 561-562 and 589-592**

While this study is limited by the lack of data saturation, the insights it provides remain valuable for informing best practices in community health. The findings reiterate well-documented barriers to achieving UHC at the community level in Kenya, particularly the challenges of stigma, misinformation, resource constraints, and public trust in government policies (38).

Future research should aim to address the study’s limitations by employing broader methodologies across multiple counties to enable more generalizable and robust conclusions. A comparative study involving diverse regions and larger sample sizes would provide deeper insights into CHV experiences and inform more targeted policy interventions.

## **Conclusion, line 603-606**

While the CHVs in Kiambu County embraced their role in the fight against the pandemic, they felt that they lacked support from both the government and the community. This perspective is tied to their stigmatization, experience of resource constraints that hindered their ability to safely and effectively carry out additional duties during COVID-19, lack of recognition, and a perceived sense of abandonment. The government has recently renewed its commitment to community-level healthcare service delivery under the guise of “prevention and promotion,” but continued efforts are needed to ensure that the necessary resources for the full execution of the Community Health Strategy are made available. This will be crucial in advancing a community-based approach to achieving UHC. However, given the limitations of this study, particularly the small number of FGDs and the lack of data saturation, future research should build upon these findings to provide a more comprehensive understanding of CHVs' experiences and inform stronger policy interventions.
